# In Search of Effective Anticancer Agents—Novel Sugar Esters Based on Polyhydroxyalkanoate Monomers

**DOI:** 10.3390/ijms22137238

**Published:** 2021-07-05

**Authors:** Wojciech Snoch, Dawid Wnuk, Tomasz Witko, Jakub Staroń, Andrzej J. Bojarski, Ewelina Jarek, Francisco J. Plou, Maciej Guzik

**Affiliations:** 1 Jerzy Haber Institute of Catalysis and Surface Chemistry, Polish Academy of Sciences, Niezapominajek 8, 30-239 Kraków, Poland; wojciech.snoch@ikifp.edu.pl (W.S.); tomasz.witko@ikifp.edu.pl (T.W.); ewelina.jarek@ikifp.edu.pl (E.J.); 2 Department of Cell Biology, Faculty of Biochemistry, Biophysics and Biotechnology, Jagiellonian University, Gronostajowa 7, 30-387 Kraków, Poland; dawid.wnuk@uj.edu.pl; 3 Maj Institute of Pharmacology, Polish Academy of Sciences, Smętna 12, 31-343 Kraków, Poland; staron@if-pan.krakow.pl (J.S.); bojarski@if-pan.krakow.pl (A.J.B.); 4 Instituto de Catalisis y Petroleoquimica, CSIC (Spanish National Research Council), Calle de Marie Curie, 2, 28049 Madrid, Spain; fplou@icp.csic.es

**Keywords:** anticancer agents, sugar esters, polyhydroxyalkanoates, fluorination, chemical modifications, melanoma, prostate cancer

## Abstract

Cancer is one of the deadliest illness globally. Searching for new solutions in cancer treatments is essential because commonly used mixed, targeted and personalized therapies are sometimes not sufficient or are too expensive for common patients. Sugar fatty acid esters (SFAEs) are already well-known as promising candidates for an alternative medical tool. The manuscript brings the reader closer to methods of obtaining various SFAEs using combined biological, chemical and enzymatic methods. It presents how modification of SFAE’s hydrophobic chains can influence their cytotoxicity against human skin melanoma and prostate cancer cell lines. The compound’s cytotoxicity was determined by an MTT assay, which followed an assessment of SFAEs’ potential metastatic properties in concentrations below IC_50_ values. Despite relatively high IC_50_ values (63.3–1737.6 μM) of the newly synthesized SFAE, they can compete with other sugar esters already described in the literature. The chosen bioactives caused low polymerization of microtubules and the depolymerization of actin filaments in nontoxic levels, which suggest an apoptotic rather than metastatic process. Altogether, cancer cells showed no propensity for metastasis after treating them with SFAE. They confirmed that lactose-based compounds seem the most promising surfactants among tested sugar esters. This manuscript creates a benchmark for creation of novel anticancer agents based on 3-hydroxylated fatty acids of bacterial origin.

## 1. Introduction

According to statistics provided by the World Human Organization, cancer is one of the deadliest illness globally. It is estimated that it caused 9.6 million deaths, or one in six deaths, in 2018. Lung, prostate, colorectal, stomach and liver cancers are the most common types of cancer in men, while breast, colorectal, lung, cervical and thyroid cancers are the most common among women [1]. Based on ‘Cancer Treatment and Survivorship Statistics, 2019’, the most prevalent cancers in 2019 in the USA were prostate (3,650,030), colon and rectum (776,120), skin melanoma (684,470) among males and breast (3,861,520), uterine corpus (807,860), and colon and rectum (768,650) among females [2]. Moreover, prostate cancer was the fifth most common cancer in Europe in 2018 (450,000) [3]. These statistics bring us to conclusions that searching for new solutions in melanoma and prostate cancer treatment is still essential.

Nowadays, mixed, targeted and personalized therapies are becoming more commonly used to treat cancer [2]. Many of them are combinations of surgery and radio-, chemo-, immune-, photo-, thermo- and cryotherapies. Some medicines disrupt cell division by interfering with the cell’s cytoskeleton (tubulin, mitotic spindle). Others interact with the DNA (e.g., cis-Pt, doxorubicin, fluorouracil) [4,5] of the cells or even disturb signalization by interacting with transmembrane hub proteins to drive the cell to apoptosis [6]. On the other hand, the most prospective therapies are inspired by nature itself: these are either based on monoclonal antibodies or programmed bacterial pathogens specifically targeting tumor cells within a host body [7]. In spite of the wide range and high effectiveness of the mentioned therapies, sometimes even they are not sufficient. Moreover, very often they are too expensive for common patients. Therefore, our attention was drawn to other tools that could both reduce the doses of drugs and the amount of drugs used in a given mixed, targeted therapy. The properties of sugar fatty acids esters (SFAE) make them promising candidates to be another valuable tool [8,9].

SFAEs are compounds widely used in the cosmetics and food industries because their antifungal and antibacterial properties. The physiochemistry of these surfactants allows for the formation of micelles or emulsions, which extends their applicability also to the pharmaceutical industry [10,11,12]. It has been shown that SFAE molecules are able to block glycolysis [13]. On the other hand, fatty acid chains of SFAE may cause their non-specific anchorage in cell membranes, consequently leading to the damage of transmembrane proteins, leakage of valuable substances outside of the cell and penetration of the unfavorable ones [14]. This aspect is widely discussed in the literature on the example of the influence of SFAE on microorganisms: *Bacillus* sp., *Staphylococcus* sp., *Escherichia* sp., *Salmonella* sp. or *Listeria* sp. [12,13,14]. However, activity of these surfactants may be different while investigated on mammalian cells biology [15].

In 1970s scientists began research on anti-cancer properties of SFAE [16]. The experiments carried out on both in vitro and in vivo cell models confirm that SFAE may inhibit the secretion of TNF-α and some proinflammatory cytokines such as IL-1B, IL-6 and IL-8 [17]. Moreover, their ability to inhibit in vitro excessive proliferation of bone marrow cells in the acute myelogenous leukemia model was also described [18]. It has also been shown that biological activity of SFAE may depend on the length of an aliphatic chain and their number in the whole ester molecule (mono- vs. di- vs. tri-/poly-esters). Furthermore, the type of sugar that builds SFAE plays a significant impact on their properties, affecting the hydrophilic–lipophilic balance (HLB) and thus the physical properties of the whole ester (solubility, micelles formation, stabilization of emulsion systems) [15].

The biological activity of SFAEs can also be altered by their structural modifications [19]. The literature reports that the biological activity of commonly used anti-cancer drugs can be improved through the introduction of halides, and a similar strategy can also be applied to sugar esters [20]. The most commonly used in pharmacology, and simultaneously, the most promising modifications of moieties in terms of their antiproliferative properties are perfluorination [21], chlorination [22], bromination [23] and the introduction of halogenated alkyl (trifluoromethyl, pentafluoroethyl) [24] and fluorophenyl [25] or trimethoxyphenyl [26] groups. They can be obtained by the substitution of hydrogen atoms in the carbon chain or hydroxyl groups of a sugar for halide atoms into the molecular structure. As the literature reports, the cytotoxicity of the modified molecules may be changed significantly both by the number and the location of the introduced halides. For example, substitution of all carbon atoms with 6–19 fluorine atoms in the hydrophobic part of SFAE showed promising anticancer potential. However, these compounds were also highly toxic to normal cells [27]. Therefore, it is essential to pay attention to increasing selectivity towards cancer cells without harming the native ones during the drug designing process.

**Figure 1:**
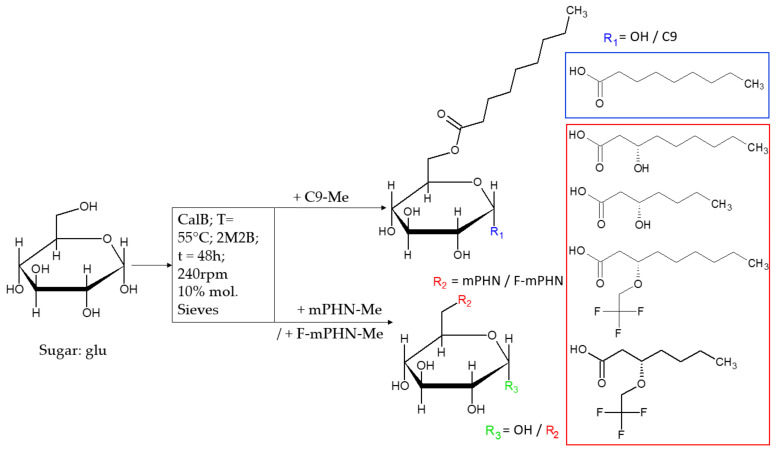
Scheme of sugar fatty acid esters enzymatic synthesis with glucose as an example. The remaining sugar-based SFAEs (galactose and lactose) and all structures of the obtained compounds are presented in Supplementary Figure S1. Where: R_1_: -OH group or nonanoic acid—structure shown in blue rectangle; R_2_: modified or not modified (mPHN or F-mPHN) PHN monomeric residues—their structures are shown in red rectangle; R_3_: -OH group or one of residues which structures are shown in red rectangle.

Here, we propose the use of bacterially derived natural monomers, namely (*R*)-3-hydroxyacids originating from polyhydroxyalkanoates polymers (PHA), as a basis for the synthesis of modified SFAE (Figure 1). PHAs can serve as material for manufacturing of nontoxic implants and other medical devices. They also are often used as media for drug delivery upon implantation [28,29,30]. To date, more than 150 different (*R*)-3-hydroxyacids have been reported, thus creating endless possibilities for further modifications [31]. Despite the differences, all of them share two unchanging features—a presence of carboxylic and a β-carbon-located hydroxyl groups. The first one enables the synthesis of a wide range of SFAEs, the latter, on the other hand, enables the introduction of various moieties into the SFAE molecule. Recently, we have reported routes towards synthesis of (*R*)-3-hydroxyacid- based glucose esters with fluorine modified analogues [29]. Here, we expanded the glucose esters library with galactose and lactose SFAE versions and tested the whole collection of compounds against human melanoma and prostate cancer cells in vitro. In this paper, we present the description on biocatalytic synthesis of novel SFAEs and their structural characterization by infrared and NMR spectroscopies combined with mass spectrometry analysis. Furthermore, we demonstrate a set of biological studies elucidating sugar esters’ biological potential. Firstly, the cytotoxicity of these novel compounds was examined, together with their metastatic properties, in concentrations below IC_50_. The results were compared to sugar esters from literature and some compounds commonly used in clinical oncology.

## 2. Materials and Methods

### 2.1. Preparation of Unmodified and Modified Polyhydroxynonanoate Monomers

Polyhydroxynonanoate (PHN) was produced using *Pseudomonas putida* KT2440 in a controlled continuous fermentation process as described previously [30]. Briefly, nonanoic acid was used as a source of carbon and energy for bacteria. The polymer was extracted with ethyl acetate and characterized as described in Sofinska et al. [30]. Next, it was decomposed to monomers through acidic methanolysis. The hydroxylated acid methyl esters were analyzed by gas chromatography. Modification of the resultant methyl esters of monomers was conducted as described previously [29]. The obtained monomers were converted into their acidic forms using *Candida antarctica* lipase B under aqueous conditions to obtain sodium salts.

### 2.2. Synthesis of Sugar Fatty Acid Esters (SFAE)

Enzymatic reactions were performed in 2-methyl-2-butanol (2M2B). Sugar substrates: lactose, glucose and galactose were supplemented with solvent and the remaining reagents, giving 20 mg mL^–1^ (2 molar equivalents) of final concentration in a reactor. The remaining substrates were: nonanoic acid methyl esters (C9) 6.04 mg mL^–1^ PHN monomer methyl esters 9.48 mg mL^–1^ and fluorinated PHN methyl esters 9.48 mg mL^–1^ (up to 1 molar equivalent), respectively. Additionally, 100 mg mL^–1^ of activated molecular sieves (4 Å) were added to maintain anhydrous conditions. The reactions were initiated by the addition of 40 mg mL^–1^ catalyst: enzyme Novozym *Candida antarctica* lipase B (CalB) and conducted at 55 °C for 48 h with shaking (240 rpm; New Brunswick^TM^ Scientific Exella E 24 Incubator Shaker Series, Eppendorf, Hamburg, Germany).

### 2.3. HPLC Analysis

Analyses were performed using UHPLC measurements in Agilent 1290 Infinity system with automatic autosampler (Santa Clara, CA, USA) and MS Agilent 6460 Triple Quad Detector (Agilent, Singapore) equipped with Zorbax Eclipse Plus 300SB-C18 Agilent column (2.1 mm × 50 mm, 1.8 µm, Santa Clara, CA, USA). To separate the components of the reaction mixture, the column was eluted at 30 °C at a flow rate of 0.4 mL min^–1^ and developed with a gradient of elution of solvent A (water) and solvent B (methanol) as follows: 0.00 min (95% A/5% B) to 1.00 min (100% B) to 4.50 min (95% A/5% B) to 5.00 min (95% A/5% B). The interval between injections was 4.5 min. MS Agilent 6460 Triple Quad tandem mass spectrometer with Agilent Jet Stream Electrospray (ESI) interface was used in negative ion mode. Nitrogen with a flow rate of 10 L min^–1^ was used as a drying and collision gas. The drying gas temperature was set to 350 °C and the shielding gas temperature to 200 °C. The capillary voltage was set to 4000 V and the nozzle voltage to 2000 V. All compounds were monitored in scanning mode and product ions with different collision energies (5–30 eV) (m/z products in Table S1 in Supplementary Materials).Workstation MassHunter Data Acquisition 1.1 version was used to control the HPLC-MS data collection and processing.

### 2.4. Preparative HPLC

Preparative scale liquid chromatography was used to purify SFAE products. The crude reaction mixture was purified on VersaPack^TM^ C18 (SUPELCO Analytical, Bellefonte, PA, USA) preparative column in the MeOH/H_2_O system with an increasing MeOH gradient of 50 to 100%. The elution of the reagents was monitored with TLC chromatography performed on C18 Merck silica gel plates (with the same elution system as in preparative separation) and high performance liquid chromatography coupled to mass spectrometer: Waters TQD instrument in scan mode ESI: +100–1000 m/z.

### 2.5. ^1^H NMR and ^19^F NMR Spectroscopy

Twenty mg samples of each compound were dried under vacuum. Samples were analyzed on Bruker (BioSpin GmBH, Rheinstetten, Germany) in CDCl_3_.

### 2.6. IR Spectroscopy

The compounds tested were dissolved in methanol. All spectra were collected by using IR microscope Nicolet iN10 (Thermo Scientific™ part of Thermo Fisher Scientific, Madison, USA). All reflection measurements were performed on gold layer by using high-sensitivity MCT-A detector within spectral range from 4000 cm^–1^ to 675 cm^–1^, 128 scans during 25 s with normal resolution just after the solvent evaporation or immediately after application in case of volatile substances. Fully automated adjustable aperture for measuring field extraction was 150 µm × 150 µm.

### 2.7. Cell Cultures

The following cell lines were used: human prostate cancer cells from metastases to the brain DU145 (ATCC^®^ HTB-81™, Manassas, Virginia); healthy human prostate epithelial cell line as a control (PNT2; Sigma-Aldrich, Darmstadt, Germany); human melanoma Hs 294T (ATCC^®^ HTB-140™, Manassas, Virginia); human epidermal keratinocytes (HaCaT) HEK001 (ATCC^®^ CRL-2404™, Manassas, Virginia); and normal human skin fibroblasts BJ (ATCC^®^ CRL2522™) as controls. The cells were cultured in 37 °C with humidified atmosphere enriched with 5% CO_2_ in appropriate cell culture media: DMEM F12Ham (Sigma-Aldrich, Darmstadt, Germany) + 5% FBS (Thermo Fisher Scientific; Gibco; Waltham, MA, USA); RPMI-1640 (Sigma-Aldrich, Darmstadt, Germany) + 10% FBS (Thermo Fisher Scientific; Gibco; Waltham, MA, USA); DMEM high glucose (Sigma-Aldrich) + 10% FBS (Thermo Fisher Scientific; Gibco; Waltham, MA, USA); DMEM low glucose (Sigma-Aldrich, Darmstadt, Germany) + 10% FBS (Thermo Fisher Scientific; Gibco; Waltham, MA, USA). All media contained a penicillin/streptomycin (Sigma-Aldrich, Darmstadt, Germany) cocktail.

### 2.8. MTT Cytotoxicity Assay

For the determination of the cytotoxic effect of tested compounds, normal (control) and cancer cell lines cells were tested at a concentration of 2.5 × 10^4^ cells cm^–^^2^ in appropriate culture medium into 96 well microplates (VWR, Radnor, PA, USA). Cells were preincubated with four different concentrations of tested compounds: 0.5, 0.25, 0.125 and 0.0625 mg mL^–1^ for 24 h, 72 h and 120 h in cell incubator. After the incubation, MTT labelling reagent (final concentration 0.5 mg mL^–1^) and solubilizing solution were added according to the protocol. The samples absorbance was measured using a microplate reader (Multiscan FC; Thermo Fisher Scientific, Waltham, MA, USA) by 570 nm wavelength, 620 nm for reference.

### 2.9. Fluorescent Staining

Cellular studies 12-well plates were used, cell density 3 × 10^5^ per well. After 24 h and 48 h of incubation with tested compounds (0.0625 mg mL^–1^), the samples were fixed according to standard protocol. Tubulin (green; AlexaFluor-488 mouse anti-β-tubulin IgG; BD Biosciences, San Jose, CA, USA, Pharminogen; cat. 558605), vimentin (infra-red; I: rabbit-anti-vimentin IgG; GeneTex, Irvine, CA, USA; GTX100619; II: chicken-anti-rabbit-AlexaFluor647 IgG; ThermoFisher, Waltham, MA, USA) and counterstained nuclei (blue; Hoechst 33258; Sigma-Aldrich, Darmstadt, Germany) and F-actin (red; AlexaFluor546-phalloidin; ThermoFisher, Waltham, MA, USA). Next, the samples were washed 5 times with deionized water and prepared for imaging with Fluorescent Mounting Medium (Dako Omnis, Agilent, Santa Clara, CA, USA).

### 2.10. Transmigration Assay

Transmigration assay was performed using 24-well glass-bottom plate (Eppendorf) containing microporous membranes in Boyden’s chamber (Corning; pore diameter: 8 μm; membrane diameter: 6.5 mm). Native DU145 and HTB140 cells were seeded at density of 3 × 10^5^ cells per membrane with tested compounds (0.0625 mg mL^–1^) and left for 48 and 96 h for transmigration. The transmigrating cells were fixed with 3.7% formaldehyde and counted. The result is presented as a reference to the rate of cell proliferation in the corresponding compound.

### 2.11. Cell Structures Imaging

Microscopic measurements and observations were carried out with the use of fluorescent techniques performed on Zeiss Axio Observer Z.1 microscope with LSM 710 module (Carl Zeiss Microscopy GmbH Carl-Zeiss-Promenade 10, 07745 Jena, Germany). Image acquisition, processing, deconvolution and analysis were performed using Zeiss ZEN Black software ver. 8.1.0.484 and FluoRender 2.21.0. An oil immersion 40×/NA:1.4 lens was used for observations. Parameters used for acquisition of cell morphology were: nucleus—blue channel (405 nm); microtubular network—green channel (488 nm); Actin—red channel (546 nm); vimentin—far red channel (647 nm).

## 3. Results

### 3.1. Synthesis and Modification of Polyhydroxynonanoate Monomers (mPHN)

The process of bacterial polyhydroxynonanoate (PHN) synthesis provided 130 g L^–1^ of dry biomass, which after extraction, purification and filtration, gave the desired polymer with 71% efficiency. Its composition was analyzed using gas chromatography and revealed that the PHN polymer consisted of 77.7% (*R*)-3-hydroxynonatec (R3OH-C9) and 22.3% (*R*)-3-hydroxyheptane (R3OH-C7) monomeric units. A portion of that mixture was subjected to further chemical modification. The weighed, purified and dried post-reaction mixture contained fluorinated methyl esters of mPHN monomers (mPHN-Me) (C7 and C9 chains). The actual conversion of 48.5% was determined from the average expected mass for the reaction, whose theoretical efficiency is 100%. Samples were submitted to ^1^H and ^19^F NMR analyses, which confirmed the structural modification of the PHN (*R*)-3-hydroxyacids and the synthesis of the (*R*)-3-(2,2,2-trifluoroethoxy) nonanoic a (*R*)-3-(2,2,2-trifluoroethoxy)heptanoic acids (together called F-mPHN-Me, Figure S1 in Supplementary Materials). The infrared spectroscopic observation further confirmed the successful synthesis of the fluorinated compounds. The spectra of purified samples after reacting mPHN-Me with trifluoroethyl trifluoromethanosulphonate revealed the disappearance of stretching vibrations in the range 3000–3500 cm^–1^ characteristic for free hydroxylic group. Moreover, the appearance of new peaks was observed (1465.1 cm^–1^, 1377 cm^–1^, 1261 cm^–1^), which correspond to -CF_3_ groups connected to the alkyl chain of the monomers. A similar pattern was observed by Li et al., who analyzed the infrared substrate spectrum of CF_3_–CH_2_–O–CH_3_ molecules (described and discussed in detail in the Supplementary Materials).

### 3.2. Structural Analysis of Synthesized Sugar Esters

A library of sugar esters of both nonanoic acid and (*R*)-3-hydroxyacids derivatives was created by a biocatalytic approach employing immobilized *Candida antarctica* lipase B (CalB). In addition, a series of SFAEs with aliphatic, nonanoic and heptanoic hydrophobic tails were generated as controls to all further performed experiments. The structures of the sugar esters were confirmed by ^1^H NMR and IR analyses (Figures S4–S12 in Supplementary Materials). In general, it was observed that signals between 5.25 and 0.75 ppm, originated from newly synthesized fatty acid-sugar ester, consisted of several multiplets. Signals between 1.5 and 0.75 correspond to fatty acid chains, shifts between 2.25 and 5.25 came from sugar ^1^H signals, and multiplets around 4.5 and 4.0 came from ^1^H localized on a nearby ester bond. The differences between the fatty acids, fatty acid sugar diesters and monoester spectra are subtle and can be explained more by analyzing intensity and ratio of particular signals than appearance of additional ones. All the tested SFAE specimens showed the presence of stretching vibrations at 1725–1735 cm^–^^1^ (ester bonds), stretching vibrations in the range of 3000–3500 cm^–^^1^ (sugar ring -OH groups) and stretching vibrations in the range of 2800 to 3000 cm^–^^1^ (-CH_2_ aliphatic chain). All these spectra showed great similarity to the spectrum of the reference sample, which was a commercially available standard of sucrose monolaurate.

Furthermore, an in-depth analysis performed by MS/MS measurements supports the obtained results from spectroscopic studies and allow for quantification of reaction yields. The SFAEs were analyzed in positive and negative modes after ionization in electrospray (ESI) where they formed different adducts (ESI+: [M + H]^+^; [M + Na]^+^; [M + K]^+^ and ESI-: [M − H]^−^; [M − Cl]^−^; [M − H_2_O]^−^). These specimens were fragmented to obtain structural fragments of SFAE molecules (Table S1 in Supplementary Materials). It was noticed that the obtained sugar esters after biocatalytic synthesis were mixtures of different mono and diesters (Table 1). For example, the lipase produced, on average, a 6:4 ratio of mono- to diesters when methyl esters of nonanoic or (*R*)-3-hydroxynonanoic acids were used. Interestingly, when a fluorinated version of C9 PHN monomer was used, this proportion had changed—we observed that CalB produced 86.6% monoester with C9 PHN monomer, virtually no C9-C9 PHN diester and 12.7% of C7-C7 PHN diester. Monosaccharides, when reacted with nonmodified PHN monomers and aliphatic fatty acid esters predominantly, produced SFAE monoesters in the presence of CalB. When bulkier, fluorinated PHN monomers were used, the lipase synthesized diesters. Subsequently, the product ions with the highest intensities obtained from SFAE fragmentation in MS/MS experiments were used for quantification of the reaction yields. In general, the conversion yields obtained varied between 10.25 and 42.49% (Table 1). As a rule of thumb, glucose appeared to be the most reactive sugar in our experimental setup. We have observed that the introduction of a fluorinated moiety on a PHN monomer resulted in an averaged 2.3-fold drop in the reaction yield for monosaccharides but did not affect synthesis of lactose esters as much (1.1-fold decrease). All of these characterized SFEA mixtures were submitted for biological studies.

### 3.3. MTT Assay Indicating Anti-Proliferative Properties of C9, mPHN and F-mPHN Based SFAE

To understand anti-cancer effect of the sugar fatty esters, in vitro inhibitory concentrations (IC_50_) of each ester in the MTT test were determined. The experiments indicated a low cytotoxicity of the referring compounds which the hydrophilic SFAE groups are made of, namely, glu, gal and lac (data not shown). Similarly, the toxicity of the SFAE’s hydrophobic molecules (i.e., nonanoic acid and its sodium salt and modified and unmodified PHN monomers (either in acidic or salt forms)) oscillated in the range of 0.23 to over 4.09 × 10^−3^ mol L^–^^1^, respectively. They were two- to fourfold higher than the IC_50_ of the tested mPHN-based sugar esters. The most promising group of the potential therapeutic compounds turned out to be the esters of the PHN monomers armed with trifluoroethyl groups and also mPHN lactose ester (Table 2 and Supplementary Figure S15). In the case of F-mPHN-glu and F-mPHN-gal, the toxicity against DU145 prostate cancer cells was around 0.1 × 10^−3^ mol L^–^^1^ (after 72 h and 120 h) and 0.08–0.33 × 10^−3^ mol L^–^^1^ of F-mPHN-lac, respectively. For comparison, the IC_50_ values for sodium salts of the fluorinated monomers PHN alone were 0.23 × 10^−3^ mol L^–^^1^ after 24 h and >1.84 × 10^−3^ mol L^–^^1^ after 120 h. The IC_50_ values of the esters for PNT2 (control) cells were respectively: 0.93 × 10^−3^ mol L^–^^1^ for fluorinated glucose esters, 1.18 × 10^−3^ mol L^–^^1^ for galactose esters and 1.16 × 10^−3^ mol L^–^^1^ for lactose esters. Fluorinated PHN monomers alone gave this effect at more than 4.09 × 10^−3^ mol L^–^^1^ at 72 h and 1.84 × 10^−3^ mol L^–^^1^ at 120 h.

Analyzing the effect of sugar esters based on F-mPHN against the melanoma HTB140 line after 24 and 72 h incubation, a higher effectiveness of F-mPHN-glu in antiproliferative activity (IC_50_ values 0.06 and 0.09 × 10^−3^ mol L^–^^1^, respectively) than F-mPHN-gal (0.156 × 10^−3^ mol L^–^^1^) could be observed. Extending the times of incubation up to 120 h did not affect the cancer cells negatively. It caused increase of IC_50_ concentrations up to 0.37 × 10^−3^ mol L^–^^1^ for F-mPHN-glu, up to 0.25 × 10^−3^ mol L^–^^1^ for F-mPHN-gal and 0.39 × 10^−3^ mol L^–^^1^ for F-mPHN-lac, respectively. In relation to the HaCaT and HSF control cells it was necessary to use about two- to eightfold fold greater concentrations of the discussed compounds, respectively. mPHN-lac showed much higher toxicity against DU145 line than their mPHN-glu and mPHN-gal counterparts and less than those having trifluoroethyl groups. The IC_50_ value of mPHN-lac dropped from 0.16 × 10^−3^ mol L^–^^1^ at 24 h to 0.13 × 10^−3^ mol L^–^^1^ at 72 h to less than 0.09 × 10^−3^ mol L^–^^1^ at 120 h. mPHN-lac turned out to be less harmful to PNT2 cells because their IC_50_ values decreased from at least 0.42 × 10^−3^ mol L^–^^1^ after 24 h to 0.16 × 10^−3^ mol L^–^^1^ after 120 h. Additionally, a more promising situation was not the case of melanoma cells. The IC_50_ of these compounds were 0.69 × 10^−3^ mol L^–^^1^ after 24 h; 0.28 × 10^−3^ mol L^–^^1^ after 72 h and 0.43 × 10^−3^ mol L^–^^1^ after 120 h for HTB140 cells, whereas IC_50_ after 72 h for control cells: HaCaT was 0.38 × 10^−3^ mol L^–^^1^ and 0.50 × 10^−3^ mol L^–^^1^ for HSF.

### 3.4. Cell Structures Imaging Indicate Reorganization of Intermediate Filaments in the Presence of a Selected SFAE

**Figure 2:**
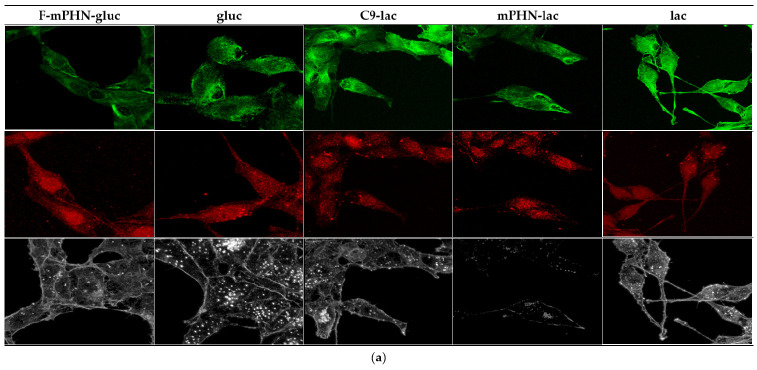

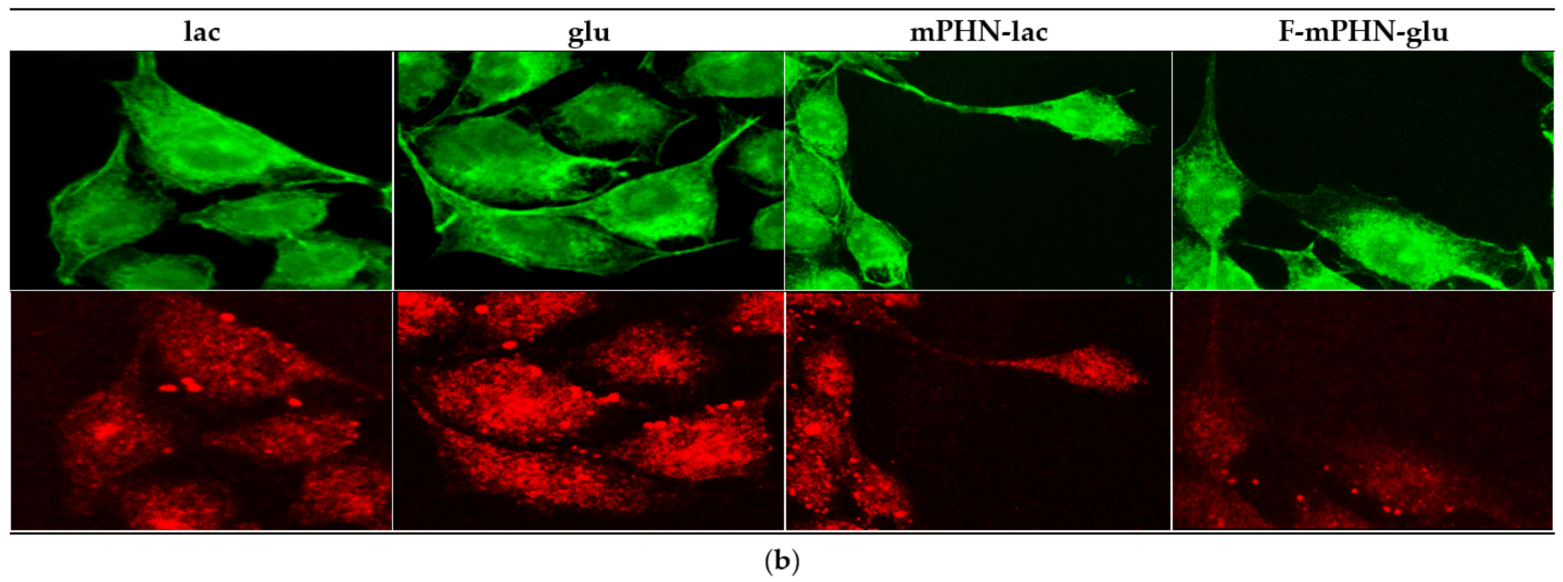

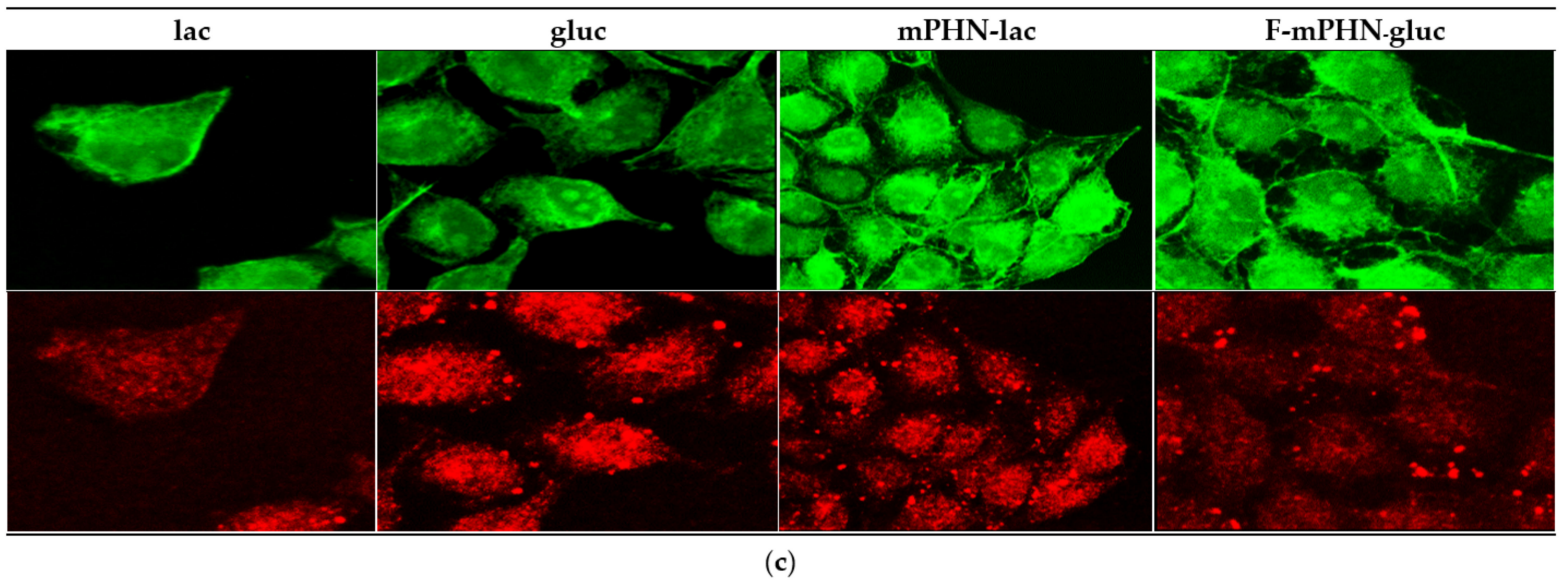
Changes in cytoskeleton of cancer cells visualized with confocal microscopy. Microtubular network—green channel (488 nm); Actin-cytokinoskeleton—red channel (546 nm); intermediate filaments (vimentin)—white channel (excitation—far red 647 nm). Brighter areas (greater density) that can be observed in green channels indicate greater polymerization of microtubules. An increased presence of biodiverse pickling inside the cytoplasm can be seen in red channels suggests an elevated content of depolymerized actin: (**a**) DU145 after 48 h of incubation; (**b**) High resolution images of DU145 after 48 h of incubation; (**c**) High resolution images of HTB140 after 48 h of incubation.

Three cytoskeletal elements were analyzed: actin filaments (red ladder), microtubules (green ladder) and intermediate filaments (vimentin—white; Figure 2). These fibers showed morphology characteristic for normal cells grown on glass [32]. The actin filaments were thick and rarely distributed and the microtubules were seen as thick and distinctly separated beams. It was also noticed (Figure 2b) that the presence of the investigated SFAE caused some changes in the other components of the cytoskeleton: actin and microtubules. In the case of microscopic images showing the cytoskeleton, an increased presence of biodiverse pickling inside the cytoplasm can be seen, which may suggest an elevated content of depolymerized actin. On the other hand, the microtubules in cells in the presence of sugar esters show a greater density of fibers and their lower diameter. Similar changes in the cytoskeleton are often associated with the apoptosis process [33,34]. The literature shows the direct influence of the cell’s environment on the shape, dynamics and architecture of both actinic and microtubule fibers [32]. However, these differences are not big enough to specify and propose any certain intracellular mechanism of interaction with the SFAE.

**Figure 3:**
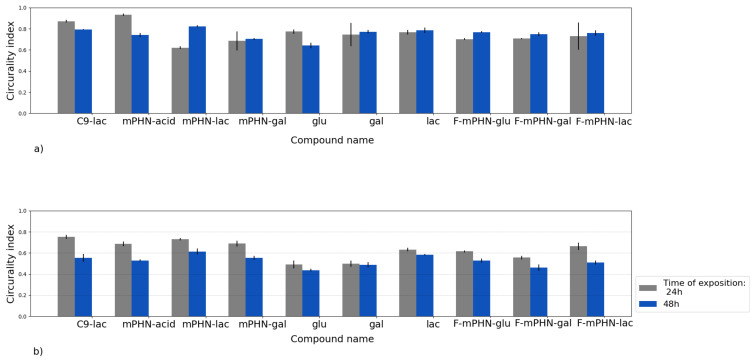
Changes of nuclei roundness based on image analysis algorithms. These small changes may indicate some reorganizations of cytoskeleton but are not typical for metastatic transition: (**a**) DU145; (**b**) HTB140.

Analysis of cell nuclei shape provided information about their condition. Using image analysis algorithms their roundness was determined for each cell individually (Figure 3). For DU145 cells (Figure 3a) after the first 24 h, the roundness of nuclei was in the range of 0.6–0.9, which is characteristic for normal cells in standard culture on glass medium [35]. After 48 h no significant changes in nuclei shapes of tested cells did not appear. Only in case of mPHN-lac was an increase in roundness was observed. For the cells cultured in C9-lac and mPHN acid, a decrease in nucleus roundness index was observed.

In the case of the HTB140 cell line, the situation was different (Figure 3b). It was clearly visible that for these cells the general circularity index of the organelle was lower and in the range 0.5–0.75, which is normal for elongated melanoma nuclei and usually associated with changes in cytoskeleton architecture and focal adhesion appearance [36]. These changes correlated with subtle alterations of in microtubules and intermediate filaments observed in microscope (Figure 2). After the first 24 h from the start of cultivation the highest circularity index was presented by cells with C9-lac and mPHN-lac. The smallest round nuclei were found in cells cultured with glucose, galactose, mPHN and F-mPHN sodium salts. The changes of the nuclei shape caused by the compounds were more significant after the next 24 h. The changes occurred in almost all cell groups and a decrease in roundness index was observed in each case. The biggest change occurred in a presence of C9-lac esters, mPHN-lac and F-mPHN-lac.

**Figure 4:**
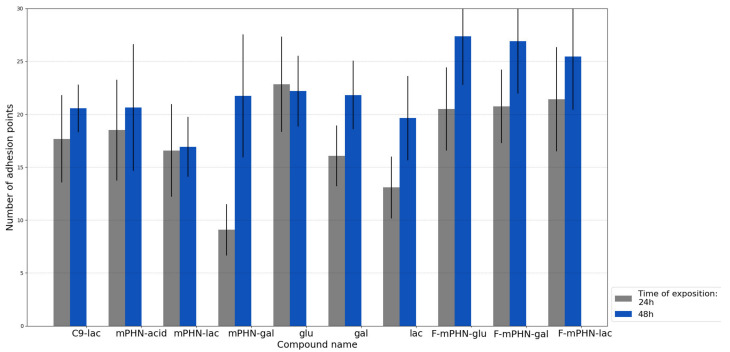
Number of adhesion points formed by DU145 cells during incubation with the investigated compounds (with concentrations below IC_50_). Adhesion points defined as dot forms placed in the focal plane located between the cell body and glass surface. Their greater number may suggest intensification of migration process caused by investigated compounds. In case of HTB140 cells, no vimentin analysis was performed due to its almost complete depolymerization which indicates strong impact of SFAE on cytoskeleton.

Subcellular structures imaging brought our attention to changes of intermediate filaments. Our study focused on the analysis of adhesion points. They were observed as dot forms placed in the focal plane located between the cell body and glass surface. After 24 h, DU145 cells formed the smallest number of adhesion points in medium supplemented with mPHN-gal and mPHN sodium salt. All cells grown in F-mPHN mediums were characterized with a high number of adhesion points similar to pure glucose. After 48 h in all samples, except those containing glucose, a significant increase in the number of adhesion points was observed. The highest increase occurred in cells grown in media containing mPHN-gal and F-mPHN-glu solutions. The cells grown in medium with F-mPHN-gal showed a similar growth. This growth did not exceed the statistical significance in the rest of experimental samples. In case of HTB140 cells, no vimentin analysis was performed due to its almost complete depolymerization and lack of possibility of correct imaging. Metastases is a process associated with the migration of cancer cells in the body. Its reduction may minimize this harmful phenomenon. An increase in the number of adhesion points (Figure 4) may indicate that selected compounds cause cancer cells to bond more strongly to the substrate or to the glass surface and thus their migration may be limited [37]. On the other hand, these observations may indicate an increasing the intensity of the attachment points production allowing the cells to migrate [38]. In order to verify which of these processes was more likely in our case, transmigration tests were carried out and proliferated cells were counted.

### 3.5. Inhibition of Transmigration Caused by C9, mPHN-lac and mPHN-glu

The transmigration test was a model experiment that was performed to illustrate the ability of cells to transmigration through a microporous membrane, where the pores mimic chinks between epithelial cells. Cancer cells are able to penetrate the tissues and migrate through the walls of blood vessels into the vascular system, which is referred to as epithelial to mesenchymal transition [39]. This process allows cells to populate new niches in other tissues, creating metastases. The key issue was to learn whether the cancer cells migrate in a medium supplemented with the investigated SFAE below the IC_50_ values, and if yes, whether they are able to proliferate actively after the transition process. Therefore, transmigration values obtained after 96 h for the cancer cells treated with tested compounds were checked. In parallel, the proliferation of cells with the tested compounds was examined.

**Figure 5:**
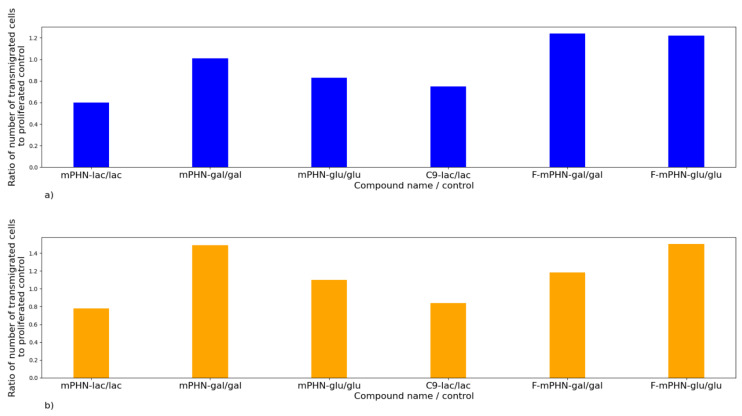
Transmigration assay of cancer cells in a presence of the investigated SFAE measured after 96 h. An increase in the ratio of proliferated cells after passing through the inserts, caused by the SFAE, may indicate that tested compounds could promote metastasis (at concentrations below the IC_50_). A decrease in this ratio may indicate lack of such effect. (**a**) DU145; (**b**) HTB140.

The results presented in Figure 5 are the ratios of transmigration to proliferation from the experimental conditions (from tested SFAE compounds at 0.0625 mg mL^–1^ concentration) in relation to the same ratios of the controls (glu, gal and lac, respectively at the same concentrations). Transmigration rates above 1.0 (100%) mean that the transmigration is enhanced, while rates below 1.0 mean that the total transmigration is inhibited by the tested compounds.

Figure 5 shows that the concentration of 0.0625 mg mL^–1^ of mPHN-lac present in the culture medium reduced transmigration of DU145 cells in 96 h by 40%, mPHN-glu ester reduced this ability by 17%, whereas C9-lac by 25% and F-mPHN-glu by 7%. The rest of compounds had no significant effect or in case of mPHN-gal and fluorinated esters even increased the cell’s ability of transmigration up to 22 to 24%. All compounds except mPHN-lac and c9-lac enhanced the transmigration of HTB140 cells from 6% (F-mPHN salt) even to 55% (F-mPHN-glu), respectively (Figure 5b). The mPHN-lac ester and the C9-lac ester decreased this factor by 22% and 16%, respectively. These data suggest that lactose esters inhibit transmigration of either prostate cancer and melanoma cells below IC_50_ concentrations. Moreover, presence of either not modified and modified mPHN component in the sugar esters affected positively on transmigration process below IC_50_ values.

## 4. Discussion

Analysis of SFAE composition basing on MS/MS detections (Table 1) confirmed the number of monomers attached to the sugar molecule impacts the quantity of alkyl halides introduced into the ester structure. The amount of hydrophobic chains attached to a sugar is determined by the lipase specificity and the conditions of the enzymatic reaction. As shown by the MTT results and reported by others, each of these modifications can have a significant effect on the cytotoxicity of the final SFAE. Synthesized sugar esters based on nonanoic acid, unmodified and modified mPHN turned out to affect DU145 and HTB140 cancer cells. The IC_50_ values determined by MTT tests were two to four times lower than in cells threated by the control compounds (C9, mPHN, F-mPHN, glu, gal and lac ranged from 0.23 to over 0.41 × 10^−3^ mol L^–^^1^). At the same time, our SFAEs were less toxic towards the healthy cell lines (PNT2, HaCAT, HSF) with the cytotoxic concentrations being eightfold higher than these used to eliminate the cancer lines (from 0.17 to 2.4 × 10^−3^ mol L^–^^1^, respectively). The range of these concentrations corresponds with the available literature data on the antiproliferative properties of other sugar esters, especially those having the same number of carbon atoms in hydrophobic part of the molecule [15].

From the experimental data gathered it was evidenced that type and quantity of the monomers attached to the sugar molecule by the lipase, and also the type of sugar used, both have impact on the biological behavior of SFAE. Based on the UHPLC-MS/MS analysis of the compositions of each synthesized SFAE, together with the corresponding IC_50_ values, the structure–toxicity relations can be discussed. For example, C9-glu and C9-lac were composed of similar ratios of monoesters to diesters but exhibited different cytotoxicity levels towards tested cancer lines. That observation may suggest that the kind of sugar used (mono- vs. disaccharide) has a greater influence on the anticancer properties of SFAE when the same hydrophobic chain is considered. A similar pattern was observed when mPHN-based SFAE were tested, however the presence of the hydroxyl group on the aliphatic chain of SFAE resulted in the increased antiproliferative character of these sugar esters. Other studies report an important correlation in the level of cytotoxicity and the chain length of the hydrophobic component [30,35]. For example, the IC_50_ of an octanoic acid glucose ester against Jurkat (Human T-cell leukemia) was 0.12 × 10^−3^ mol L^–^^1^, whereas the 16 carbon atoms counterpart was as low as 0.02 × 10^−3^ mol L^–^^1^. This suggest that in future research it will be beneficial to screen longer fatty acid mixtures originating from polyhydroxyalkanoates for their potential in modulating the cytotoxicity of synthesized SFAEs.

The introduction of trifluoroethyl groups into mPHN further increased the cytotoxicity of the glucose and galactose esters. Interestingly, this modification did not have such a great effect on the lactose-based SFAE when compared to C9-lac or mPHN-lac compounds. It is known form the literature that nonspecific modifications of the aliphatic chain of SFAE by substitutions of hydrogen atoms by halides may further reduce the biological activity of the molecule (e.g., decrease of the glucose ester’s cytotoxicity to over 0.20 × 10^−3^ mol L^–^^1^), thus suggesting that a plan for manipulation of cytotoxicity of the SFAE presented in this work is promising for potential drug designing process.

Having examined the cytotoxicity of SFAE, we looked at the subcellular structures of the assayed cell lines after their exposition to the tested compounds below their IC_50_ concentrations. Esters (especially these based on lactose) interacted to some extend with intermediate pillars, in particular with vimentin (Figure 2—white channel), which was manifested by a minor increase in the number of cluster points after 48 h exposure to all of the tested compounds (Figure 4). In the literature, there is scarce information on SFAE interaction with the cytoskeleton. However preliminary conclusions can be drawn from observations of polyethylene glycol inhibition of polymerization/depolymerization of microtubules [40] or interactions of gallic acid-based glycoconjugates that target tubulin and its colchicine binding site [41]; that may in future help to explain antiproliferative properties of PHA-based SFAE. Nuclei shape and number of adhesion points may provide information about potential metastatic stages. Therefore, we have conducted microscopic observations of these structures. There were minimal changes in the roundness of the cell’s nuclei over a period of 24–48 h that may indicate that all of the tested SFAE did not affect these organelles significantly while treating them below the IC_50_ concentrations (Figure 3). In addition, no significant differences were seen in the amounts of adhesion points between treated and untreated cells. Our survey showed decrease in transmigration of DU145 and HTB140 cells in most of the tested compounds, with the exception of these containing fluorine atoms. This phenomenon may be associated with disturbances in the cytoskeleton architecture, however it requires additional analysis of signaling pathways related to the cytoskeleton and cell movement at the molecular levels [41]. Altogether, and keeping in mind the relatively high cytotoxicity of SFAE (Table 2), we can assume that the synthesized sugar esters will not promote metastasis.

## 5. Conclusions

Despite relatively high IC_50_ values (0.06–0.17 × 10^−3^ mol L^–^^1^) of the synthesized SFAEs in comparison to commonly used chemotherapeutics, the studied compounds can compete with other SFAEs already described in the literature. Their contribution in drug designing process may rely on either using the SFAE as potentially active substances or as supporting therapeutics. These PHA-based surfactants may form microemulsions or self-microemulsifying drug delivery system (SMEDDS), which may help to stabilize and distribute commercially used medicines for cancer treatment or gene therapy; thus, SFAE-based carriers could co-work with chemotherapeutics by their own cytotoxic properties [42,43,44]. However, antimetastatic properties of our SFAE require further investigations due to their visible influence on cytoskeleton and cell intermediate filaments [41]. Finally, the panel of tested cell lines in the cytotoxicity experiments should be extended to include other lines that may be more sensitive to the synthesized compounds. In summary, the data contained within this manuscript are benchmarks for future studies related to the creation of SMEDDS based on synthetized SFAE for targeted anticancer therapies.

## 6. Patents

The work presented in this manuscript is a part of a Polish patent application number P.437233 “Use of sugar esters of fatty acids, with an acid component as a mixture of monomers derived from bacterial polyhydroxynonanoate-co-heptanoate, to inhibit tumour cell proliferation in the treatment and prevention of diseases” submitted on 8 March 2021.

## Figures and Tables

**Table 1:** Molar composition of sugar fatty acid ester based on LC-MS QQQ analysis and conversions of reactions.

Ester	Monoester with C9 Chains [%]	Monoester with C7 Chains [%]	Diester C9 Chains [%]	Diester C7 Chains [%]	Diester C9C7 Chains [%]	Conversion after 48 h [%]
C9-glu	57.8	-	42.2	-	-	17.8
C9-gal	99.9	-	-	-	-	17.8
C9-lac	56.2	-	43.8	-	-	10.4
mPHN-glu	88.1	0.7	-	-	11.2	42.5
mPHN-gal	38.6	-	18.8	42.7	-	24.3
mPHN-lac	59.4	-	40.6	-	-	12.6
F-mPHN-glu	0.4	8.5	76.0	10.3	4.6	18.9
F-mPHN-gal	-	-	-	90.9	9.1	10.3
F-mPHN-lac	86.6	0.2	0.5	12.7	-	11.0

Where ‘-’—not present.

**Table 2:** Cytotoxicity IC_50_ of SFAE determined by MTT assay.

		IC_50_ [10^−3^ mol L^–1^]								
	**Time [h]**	C9-glu	C9-gal	C9-lac	PHN-glu	PHN-gal	PHN-lac	F-mPHN-glu	F-mPHN-gal	F-mPHN-lac
DU145	24	1.32	1.56	0.92	0.93	0.75	0.16	-	-	-
	72	0.66	0.78	0.84	0.54	0.50	0.13	0.10	0.10	0.08
	120	1.32	1.56	0.46	0.32	0.30	0.09	0.10	0.10	0.34
PNT2	24	2.49	2.39	1.71	1.51	1.26	0.42	-	-	-
	72	1.11	1.23	0.92	1.31	0.61	0.20	0.93	1.18	1.16
	120	2.05	2.36	1.74	1.18	1.84	0.17	1.08	0.70	0.66
HTB140	24	1.47	1.66	0.62	1.77	0.97	0.64	0.06	0.16	0.19
	72	1.32	1.56	0.92	1.20	1.22	0.28	0.09	0.16	0.22
	120	1.13	1.56	0.81	1.69	1.50	0.44	0.37	0.25	0.39
HaCAT	72	1.32	1.56	0.92	1.43	1.20	0.38	0.73	0.58	0.64
	120	-	-	-	-	-	-	0.80	0.95	0.67
HSF	72	1.32	1.56	0.92	0.61	0.62	0.45	-	-	-
	120	-	-	-	0.76	0.50	0.63	0.76	0.50	0.63

DU145—prostate cancer; PNT2—prostate epithelium—control; HTB140—human skin melanoma; HaCAT—human skin keratynocytes; HSF—human skin fibroblasts; -—not determined. Red fillings mark the lowest concentrations needed to reduce a cell population to 50% and green—the highest. Standard deviations of IC50 values are available in Supplementary Table S2 and graphical representation contains Supplementary Figure S15.

## Data Availability

Data is contained within the article or Supplementary Materials.

## Supplementary Materials

Supplementary Materials can be found at https://www.mdpi.com/article/10.3390/ijms22137238/s1.

## Abbreviations

C9	nonanoic acid
C9-Me	nonanoic methyl ester
PHN	poly-(3R)-hydroxynonanoate-co-(3R)-hydroxyheptanoate
R3OH-C9	(3R)-hydroxynonanoate
R3OH-C7	(3R)-hydroxyheptanoate
mPHN	mixture of PHN monomers hydroxyacids: (3R)-hydroxynonanoate and (3R)-hydroxyheptanoate
mPHN-Me	mixture of PHN monomemers methyl esters: (3R)-hydroxynonanoate methyl esters
R3OH-C9-Me	(3R)-hydroxynonanoate methyl esters
R3OH-C7-Me	(3R)-hydroxyheptanoate methyl esters
F-mPHN-Me	mixture of fluorinated PHN monomers: (3R)-3-(2,2,2-trifluoroethoxy)nonanoate methyl esters and (3R)-3-(2,2,2-trifluoroethoxy)heptanoate methyl esters
glu	α-D-glucopiranose
gal	β-d-galactopyranose
lac	β-D-Galactopyranosyl-(1→4)-D-glucopiranose
C9-glu	glucose nonanoic ester
C9-gal	galactose nonanoic ester
C9-lac	lactose nonanoic ester
mPHN-glu	mixture of (3R)-hydroxynonanoate glucose esters and (3R)-hydroxyheptanoate glucose esters
mPHN-gal	mixture of (3R)-hydroxynonanoate galactose esters and (3R)-hydroxyheptanoate galactose esters
mPHN-lac	mixture of (3R)-hydroxynonanoate lactose esters and (3R)-hydroxyheptanoate lactose esters
F-mPHN-glu	mixture of (3R)-3-(2,2,2-trifluoroethoxy)nonanoate glucose esters and (3R)-3-(2,2,2-trifluoroethoxy)heptanoate glucose esters
F-mPHN-gal	mixture of (3R)-3-(2,2,2-trifluoroethoxy)nonanoate galactose esters and (3R)-3-(2,2,2-trifluoroethoxy)heptanoate galactose esters
F-mPHN-lac	mixture of (3R)-3-(2,2,2-trifluoroethoxy)nonanoate lactose esters and (3R)-3-(2,2,2-trifluoroethoxy)heptanoate lactose esters
