# Supplementary material for: In Search of Effective Anticancer Agents—Novel Sugar Esters Based on Polyhydroxyalkanoate Monomers
Source: Int J Mol Sci. 2021 Jul 5;22(13):7238. doi: 10.3390/ijms22137238 (PMC8268987; doi:10.3390/ijms22137238)
Supplement: Supplementary file 1 [file ijms-22-07238-s001.zip › ijms-1258640-supplementary.pdf]

# In search for effective anticancer agents – novel sugar esters based on polyhydroxyalkanoate monomers

*Wojciech Snoch[1], Dawid Wnuk[2], Tomasz Witko[1], Jakub Staroń[3], Andrzej J. Bojarski[3], Ewelina Jarek[1], Francisco J. Plou[4], Maciej Guzik[1]*

[1] Jerzy Haber Institute of Catalysis and Surface Chemistry, Polish Academy of Sciences, Niezapominajek 8, 30-239 Kraków, Poland

[2] Department of Cell Biology, Faculty of Biochemistry, Biophysics and Biotechnology, Jagiellonian University, Gronostajowa 7, 30-387 Kraków, Poland

[3] Institute of Pharmacology, Polish Academy of Sciences, Smętna 12, 31-343 Kraków, Poland

[4] Instituto de Catalisis y Petroleoquimica, CSIC (Spanish National Research Council), Calle de Marie Curie, 2, 28049 Madrid, Spain

* Correspondence: maciej.guzik@ikifp.edu.pl; Tel.: +48 12 6395 159

**SUPPLEMENTARY**

**Index of figures and tables:**


- Fig 1. List of compound names, shortcuts and structures generated in ChemSketch
- Table 1. Precursor and product ions of sugar esters obtained in MRM LC-MS QQQ analysis:
- Fig 2. IR spectrum of C12- (sucrose monolaurate) as a referring compound

  Fig 3. IR spectra of methyl esters of not modified PHN monomers (mixture of 3-(*R*)-hydroxynonanoic and 3-(*R*)- hydroxyheptanoic acids)
- Fig 4. Spectra analysis of methyl esters of methyl esters of 3-(*R*)- (2 -2′- 2′′- trlifluoroethyl)-nonanoic and heptanoic acids (fluorinated PHN monomers or mPHN-F)
- Fig 5. Spectral analysis of glucose nonanoate (C9-glu)
- Fig 6. Spectral analysis galactose nonanoate (C9-gal)
- Fig 7. IR spectra of lactose nonanoate (C9-lac)
- Fig 8. Spectral analysis of glucose mono and diesters esters originated from mixture of not modified PHN monomers (mPHN-glu)
- Fig 9. Spectral analysis of galactose mono and diesters esters originated from mixture of not modified PHN monomers (mPHN-gal)
- Fig 10. Spectral analysis of lactose mono and diesters esters originated from mixture of not modified PHN monomers (mPHN-lac)
- Fig 11. IR spectra of glucose mono and diesters esters originated from mixture of fluorinated PHN monomers (F-mPHN-glu)
- Fig 12. IR spectra of galactose mono and diesters esters originated from mixture of fluorinated PHN monomers (F-mPHN-gal)
- Table 2. Cytotoxicity of SFAE determined by MTT assay

- Fig 13. IR spectra of  lactose mono and diesters esters originated from mixture of fluorinated PHN monomers (F-mPHN-lac)
- Fig 14. IR spectra of fluorinated esters set together

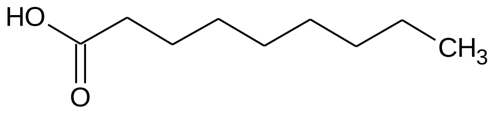


a) **C9**: nonanoic acid

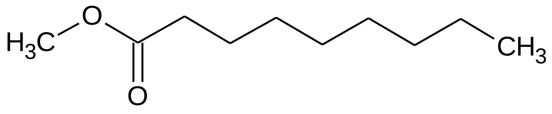


b) **C9-Me**: nonanoic methyl ester

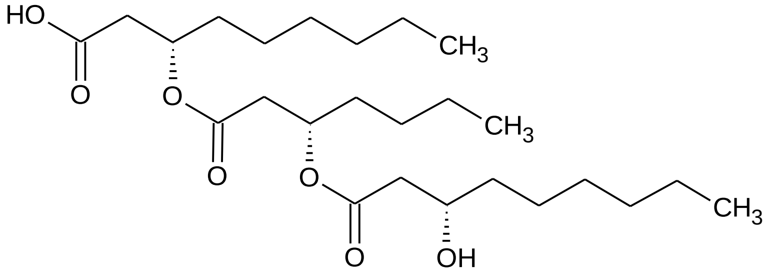


c) **PHN**: poly -(3*R*)-hydroxynonanoate- co- (3*R*)-hydroxyheptanoate

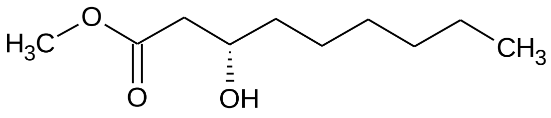


d) **R3OH-C9-Me**: mixture of (3*R*)-hydroxynonanoate methyl esters

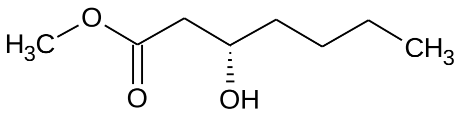


e) **R3OH-C7-Me**: mixture of (3*R*)-hydroxyheptanoate methyl esters

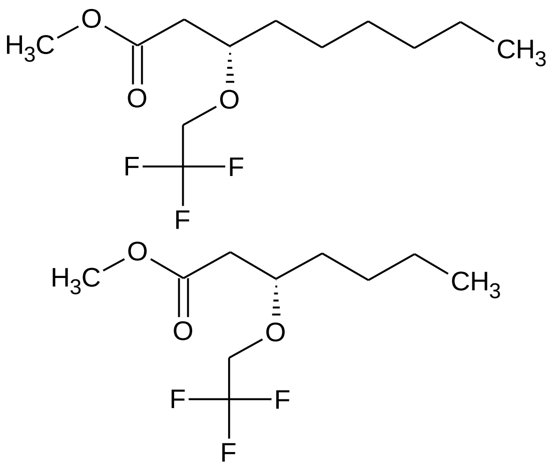

f) **F-mPHN-Me**: mixture of fluorinated PHN monomers: (3*R*)-3-(2,2,2-trifluoroethoxy)nonanoate methyl esters and (3*R*)-3-(2,2,2-trifluoroethoxy)heptanoate methyl esters

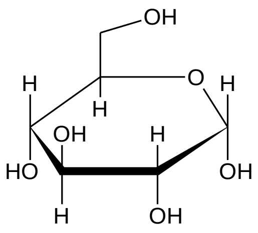

g) **glu**: α-D-glucopiranose

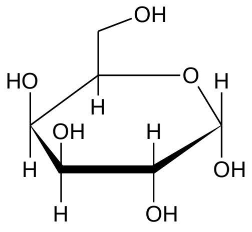

h) **gal**: β-D-galactopyranose

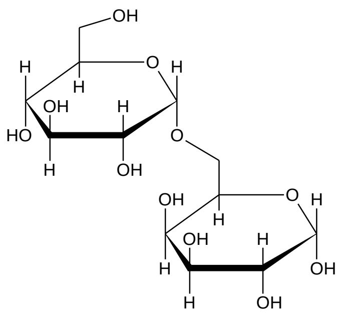

i) **lac**: β-D-Galactopyranosyl-(1→4)-D-glucopiranose

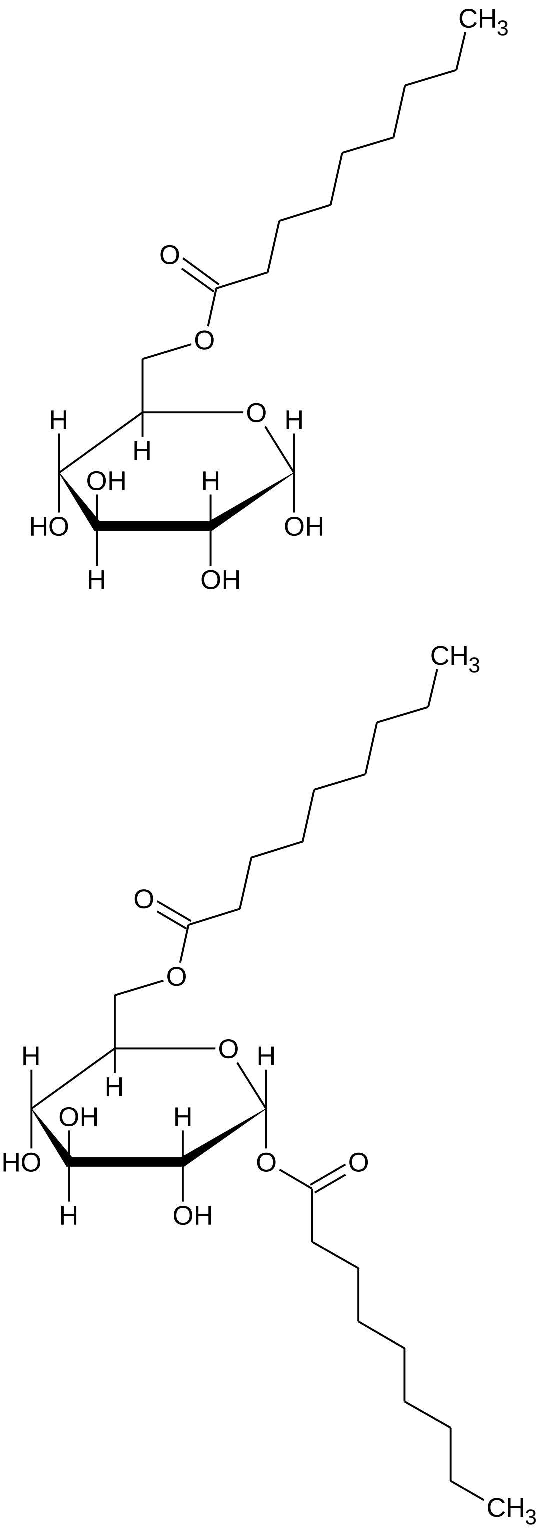

j) **C9-glu**: mixture of glucose nonanoic mono and diesters

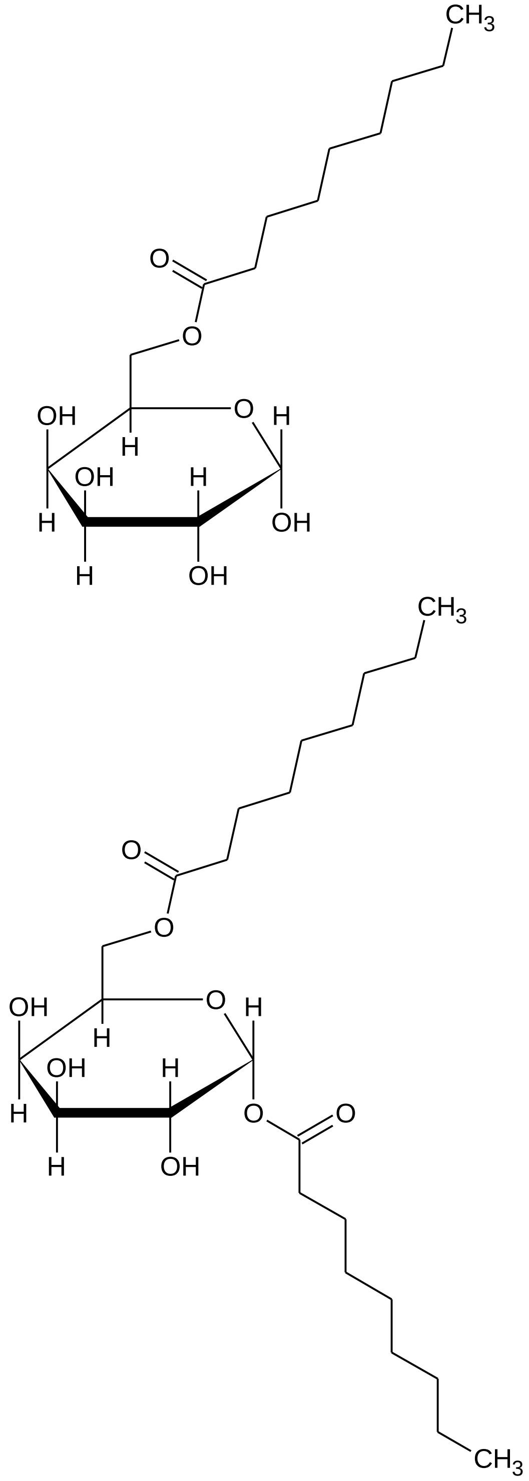

k) **C9-gal**: mixture of galactose nonanoic mono and diesters

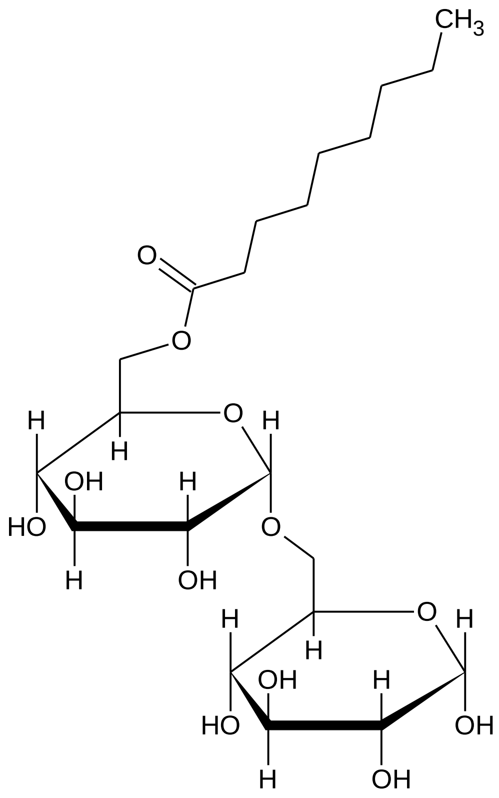

CH3
O
O
O
H
H
H
OH
H
HO
H
OH
O
O
H
H
H
OH
H
HO
H
OH
OH

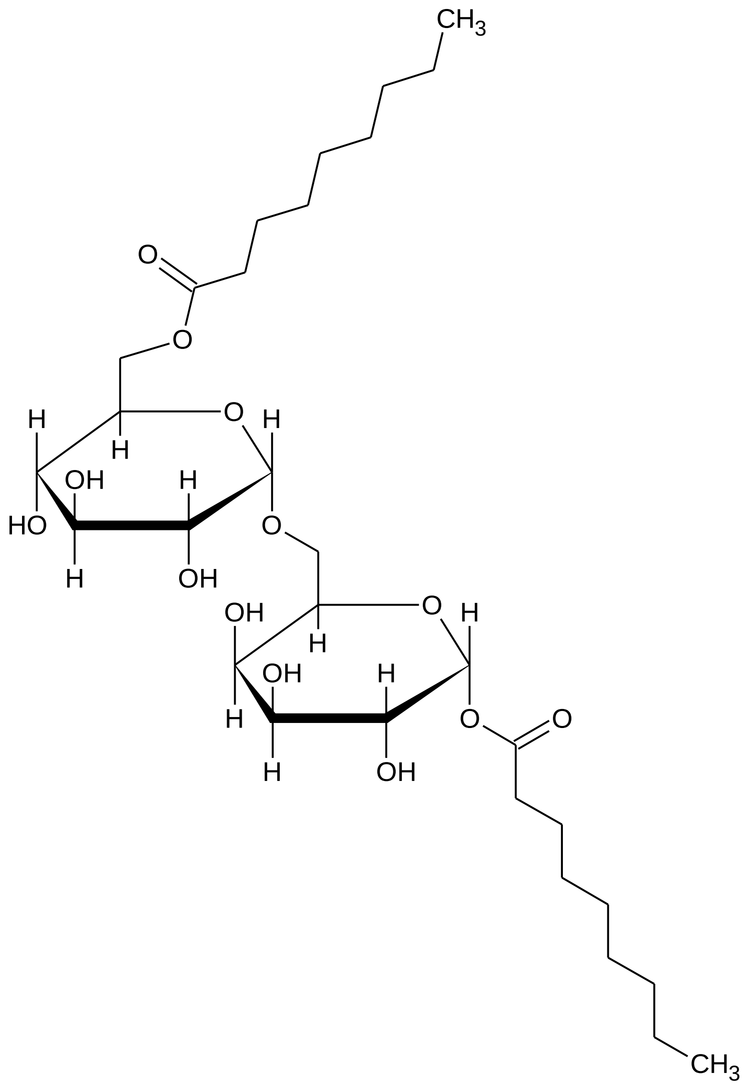


l) **C9-lac**: mixture of lactose nonanoic mono and diesters

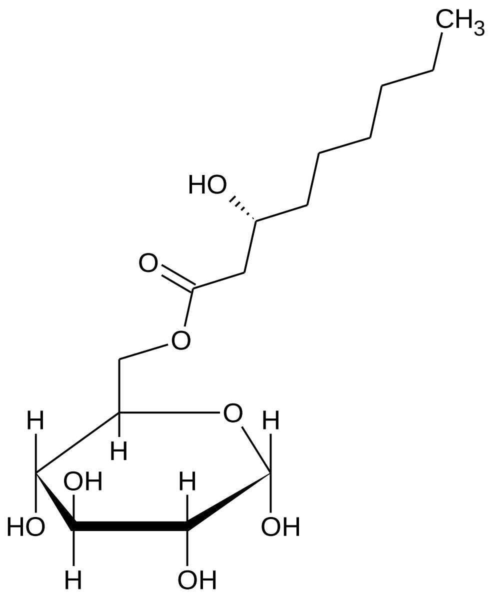
CH3
HO
O
O
O
H
H
H
OH
H
HO
OH
H
OH

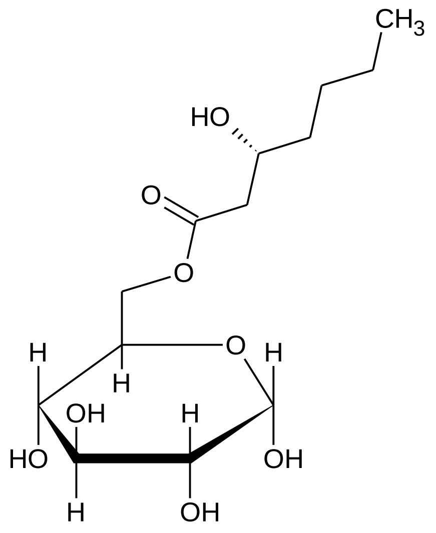
CH3
HO
O
O
O
H
H
H
OH
H
HO
OH
H
OH

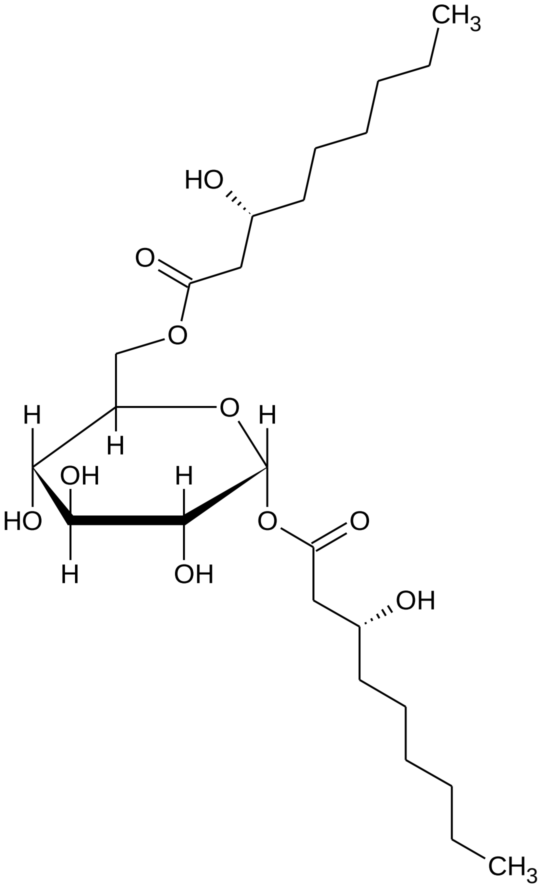

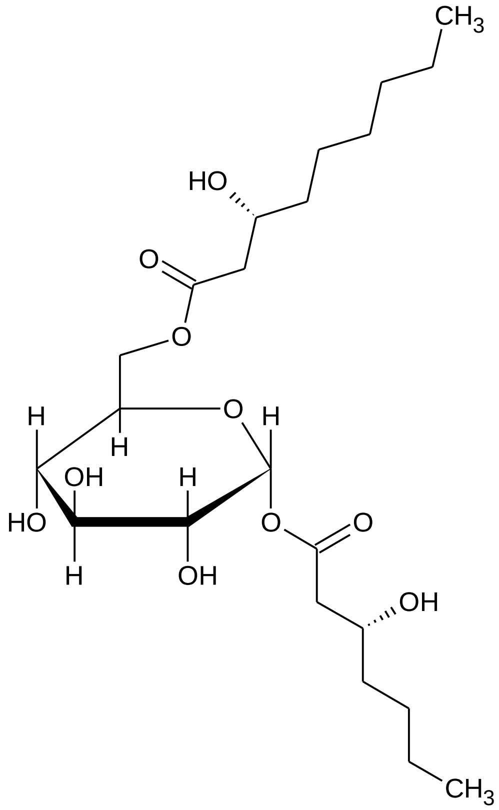

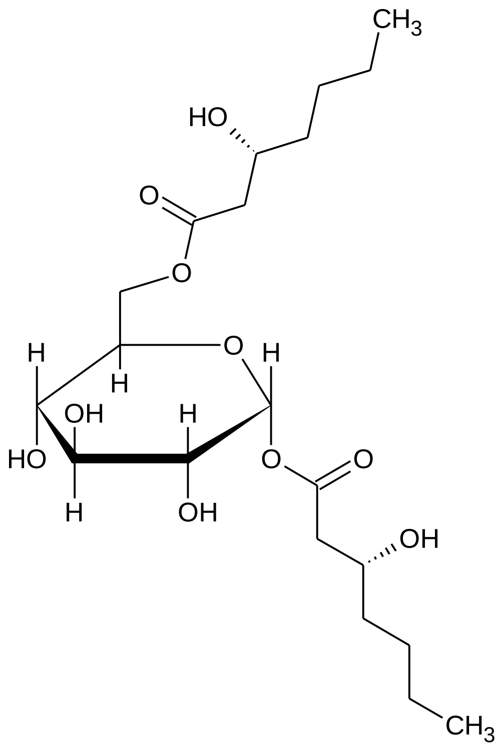


m) **mPHN-glu**: mixture of (3*R*)-hydroxynonanoate glucose esters and (3*R*)-hydroxyheptanoate glucose mono and diesters

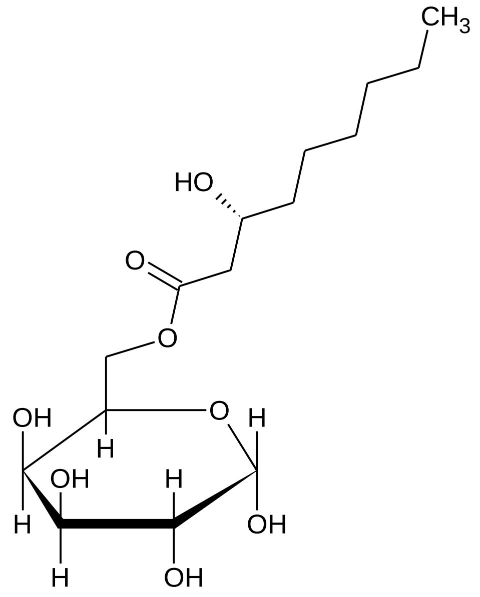

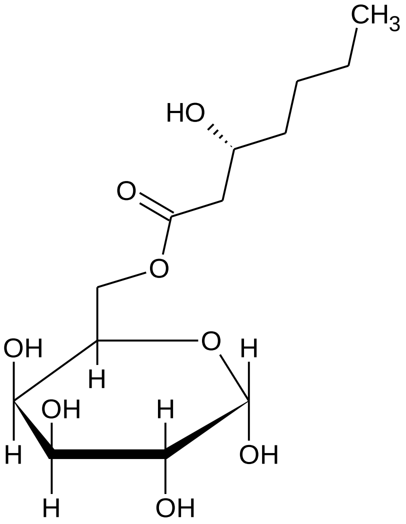

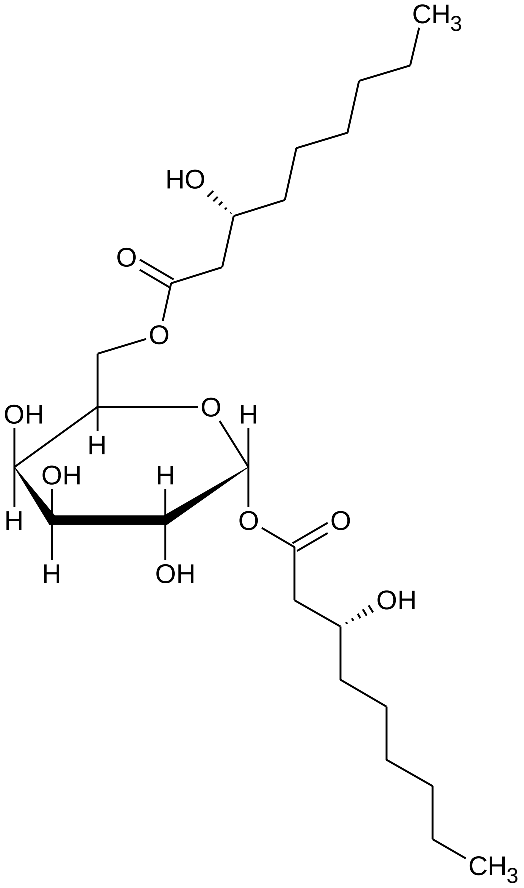

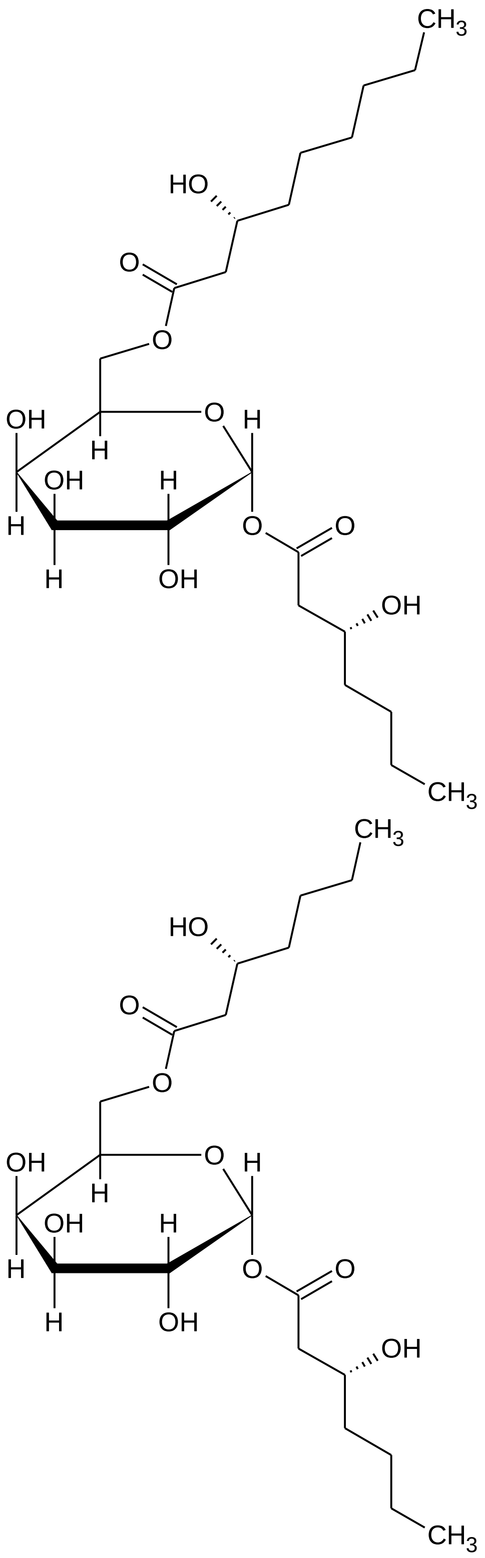

n) **mPHN-gal**: mixture of (3*R*)-hydroxynonanoate galactose esters and (3*R*)-hydroxyheptanoate galactose mono and diesters

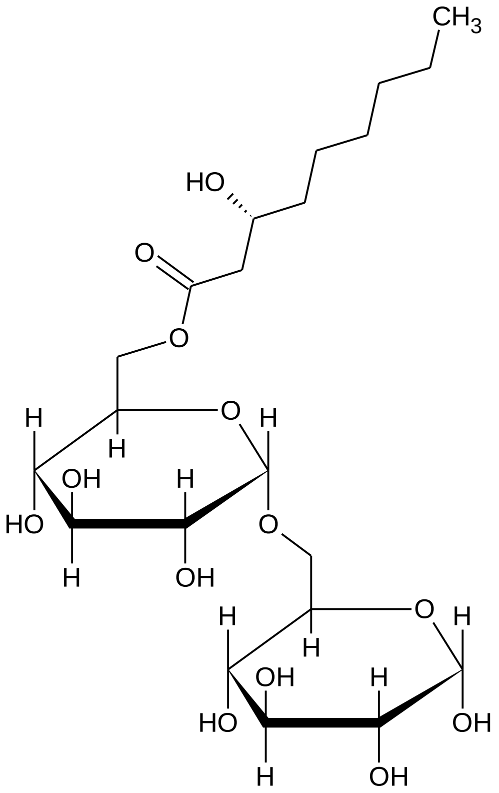

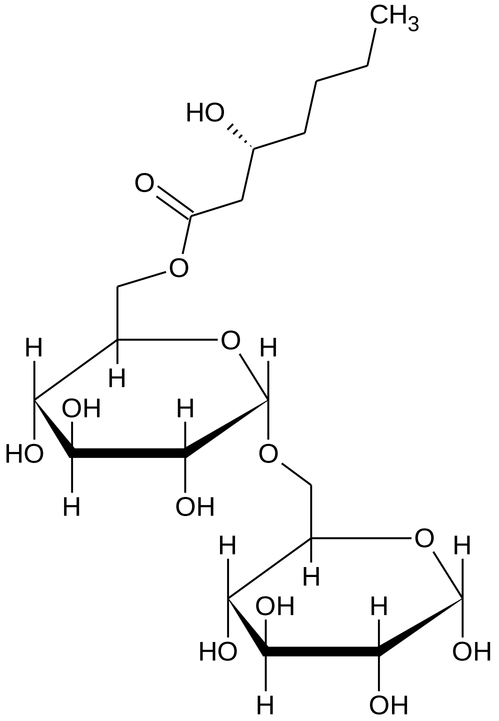

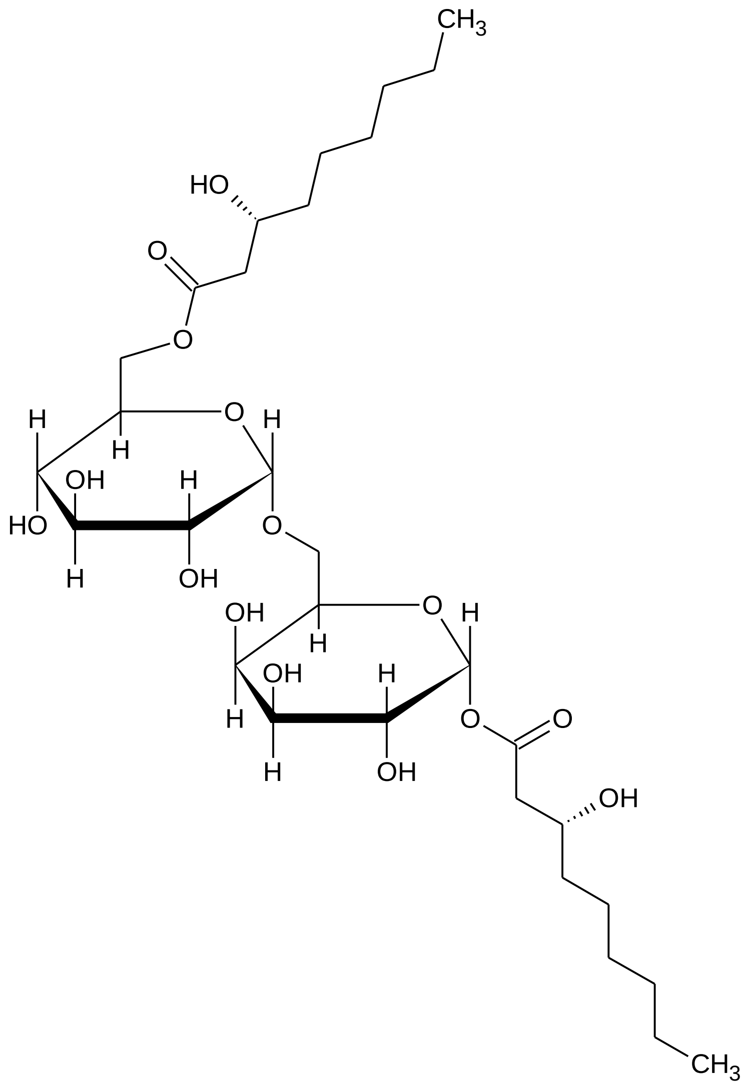

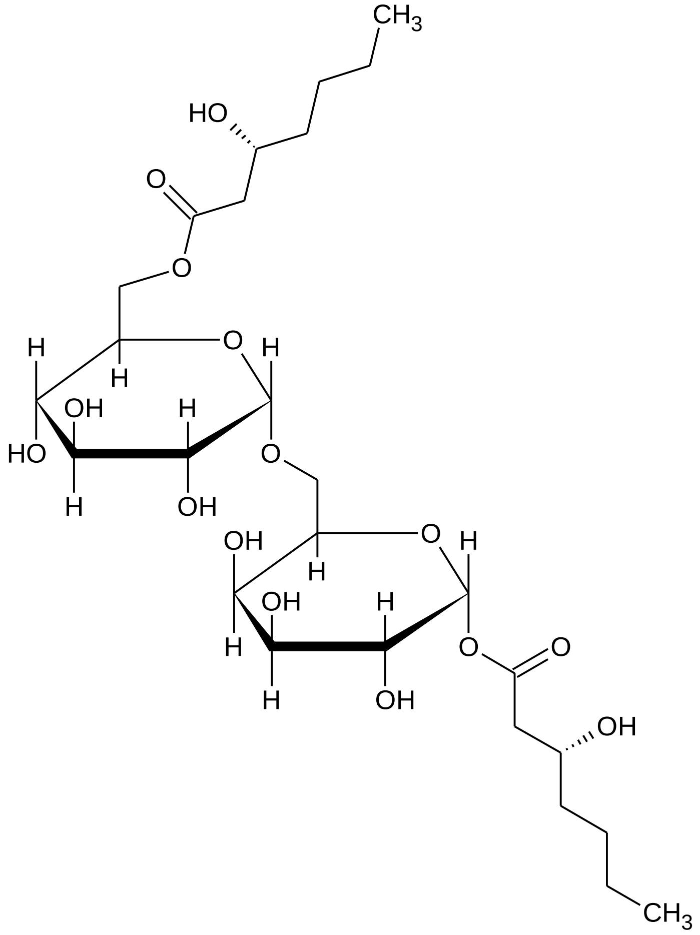

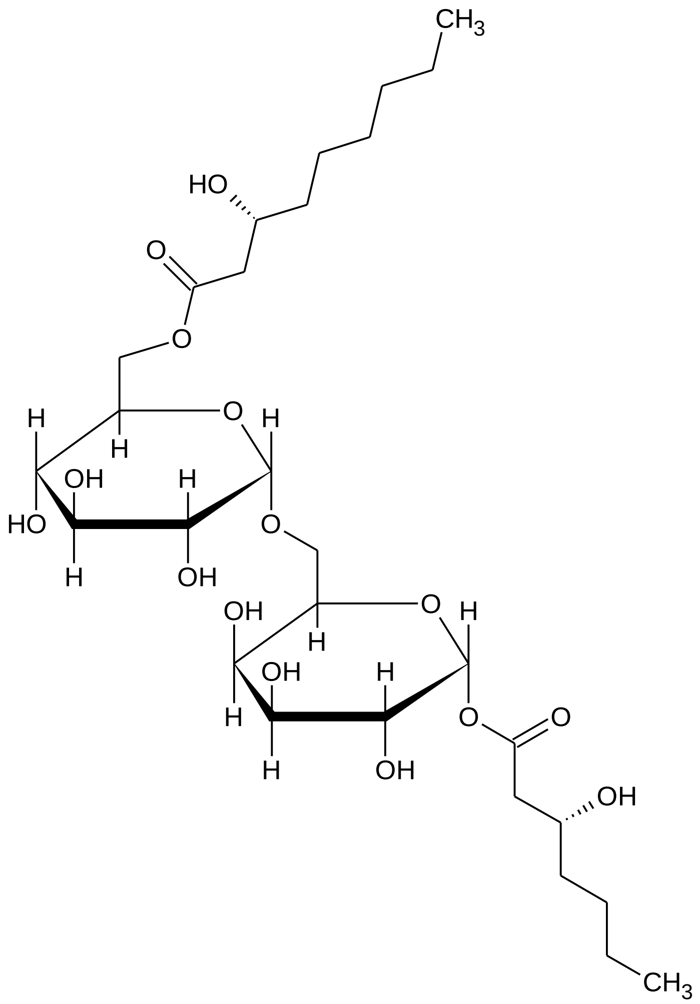


**o) mPHN-lac**: mixture of (3*R*)-hydroxynonanoate lactose and (3*R*)-hydroxyheptanoate lactose mono and diesters

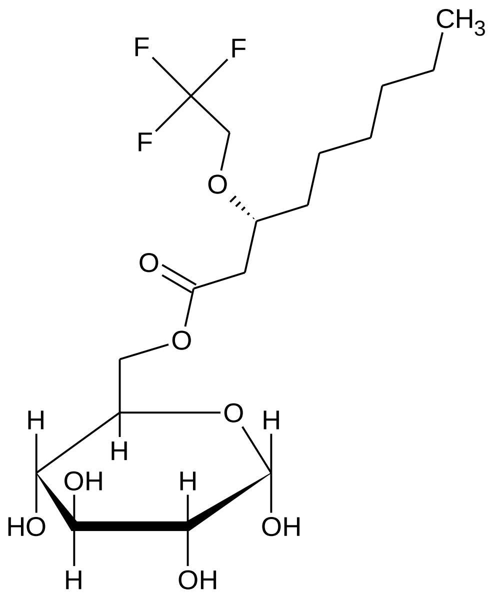
F
F
F
CH3
O
O
O
O
H
H
H
OH
H
HO
OH
H
OH

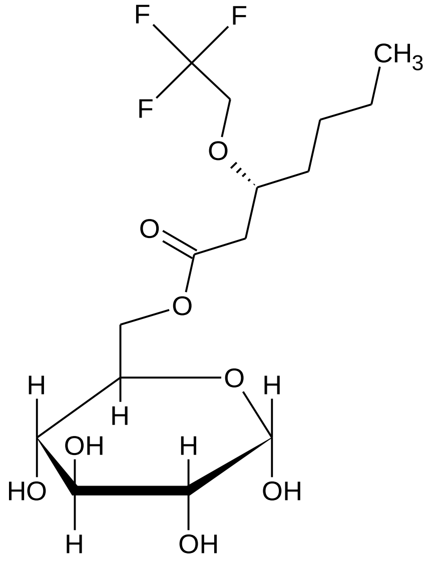
F
F
F
CH3
O
O
O
O
H
H
H
OH
H
HO
OH
H
OH

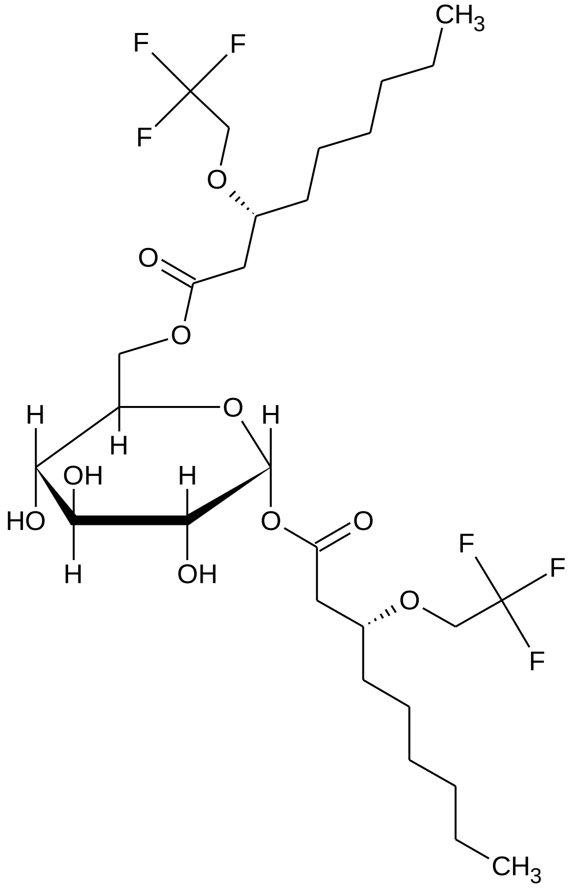

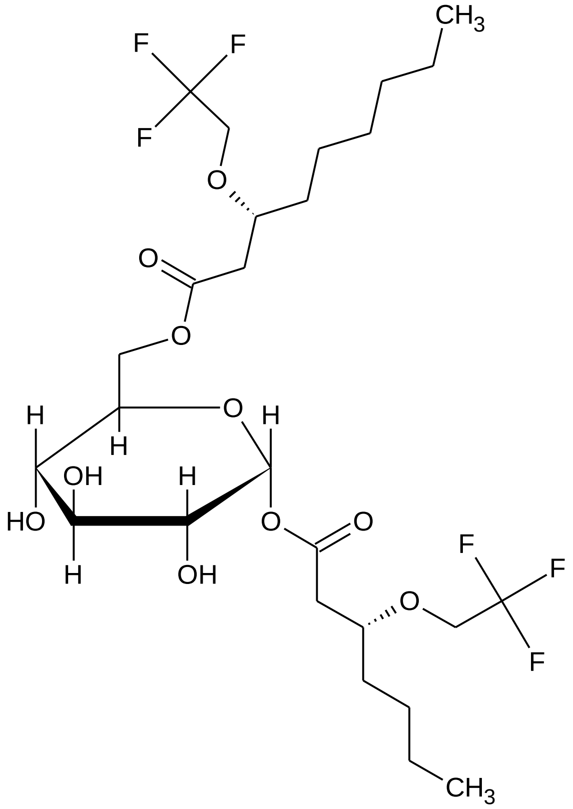

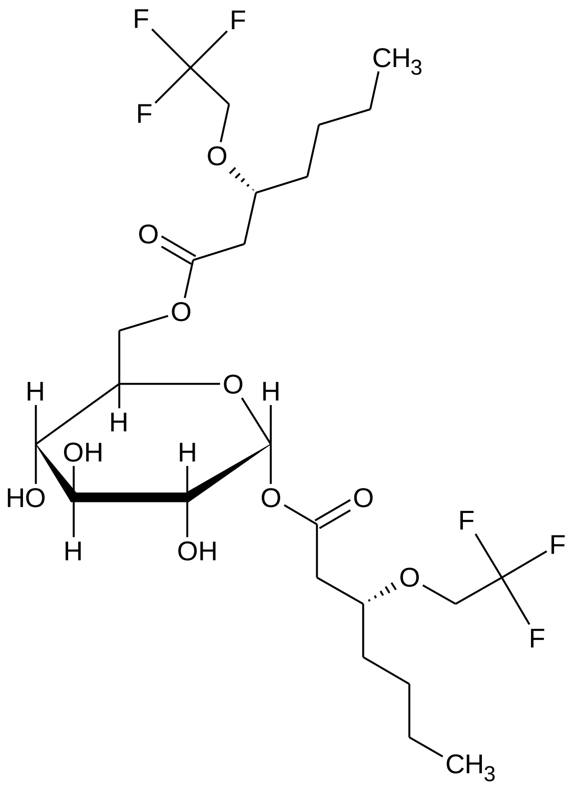


p) **F-mPHN-glu**: mixture of (3*R*)-3-(2,2,2-trifluoroethoxy)nonanoate glucose and (3*R*)-3-(2,2,2-trifluoroethoxy)heptanoate glucose mono andesters

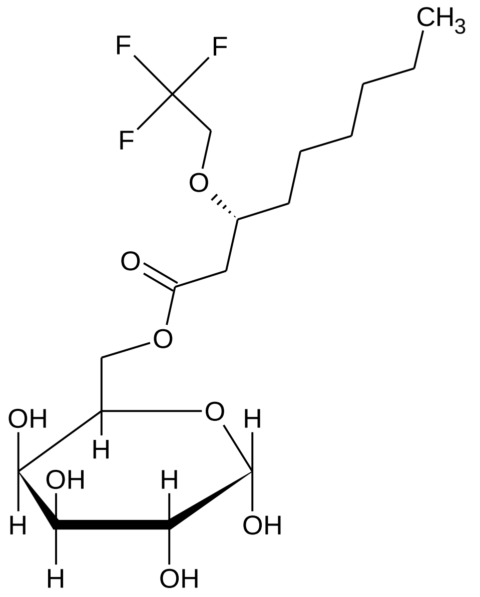

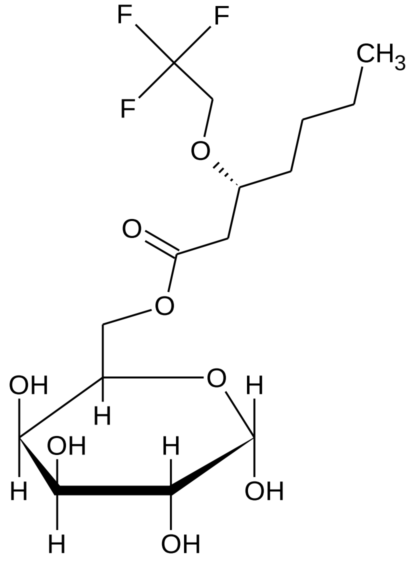

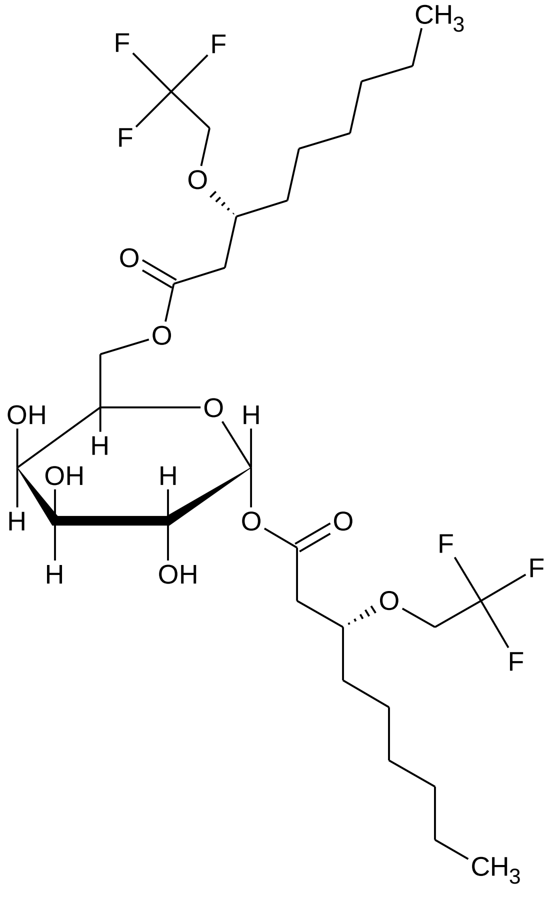

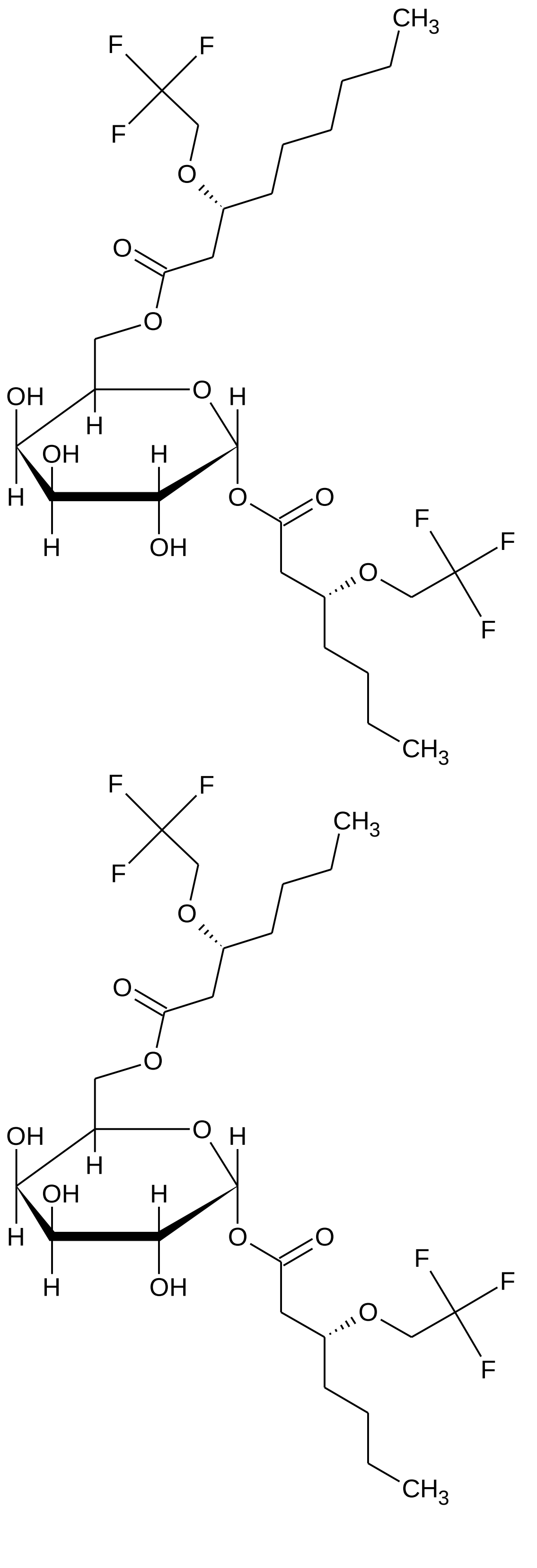

r) **F-mPHN-gal**: mixture of (3*R*)-3-(2,2,2-trifluoroethoxy)nonanoate galactose and (3*R*)-3-(2,2,2-trifluoroethoxy)heptanoate galactose mono and diesters

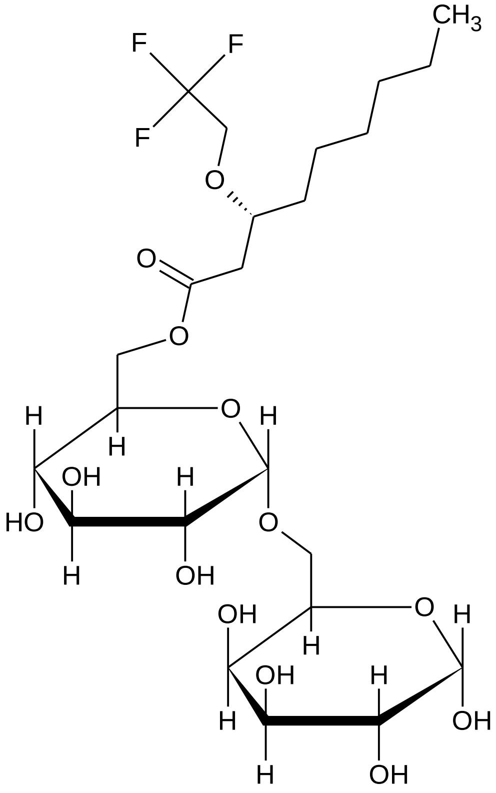

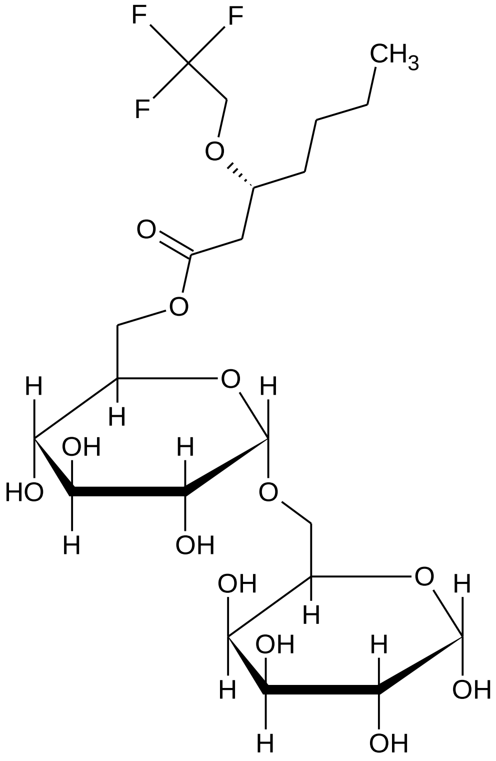

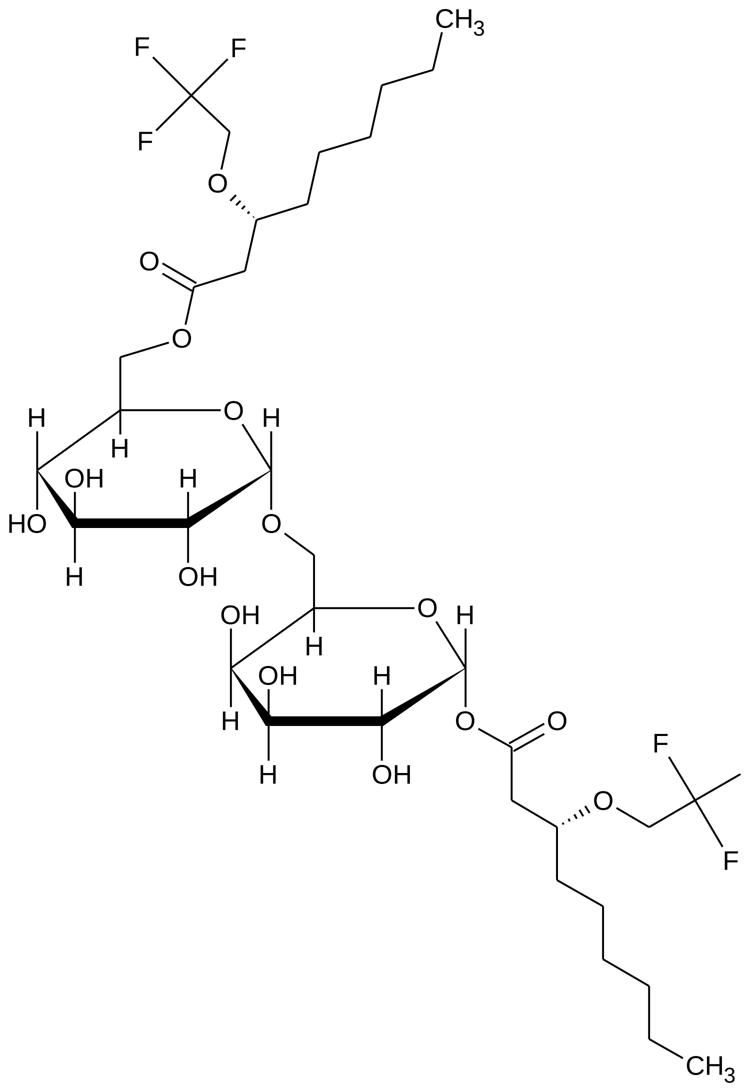
CH3
F
F
F
O
O
O
O
H
H
H
OH
H
HO
H
OH
O
OH
O
H
OH
H
OH
H
H
O
O
OH
F
F
F
CH3

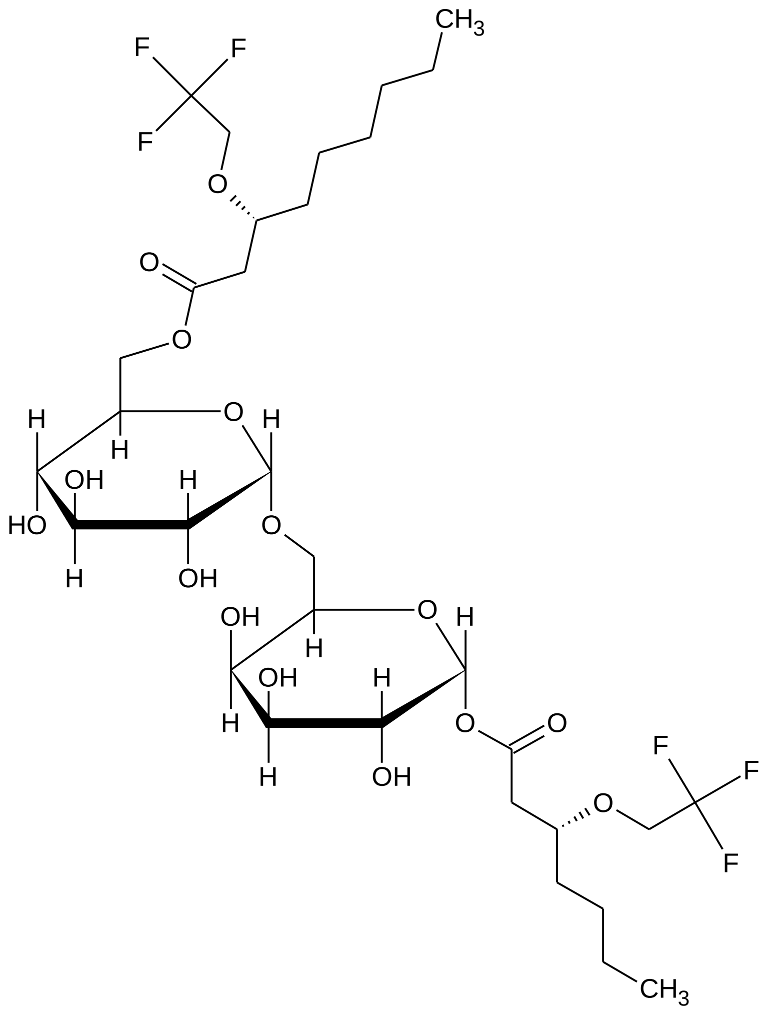

CH3
F
F
F
O
O
O
H
O
H
H
OH
H
HO
H
OH
O
OH
O
H
H
OH
H
H
H
OH
O
O
F
O
F
F
CH3

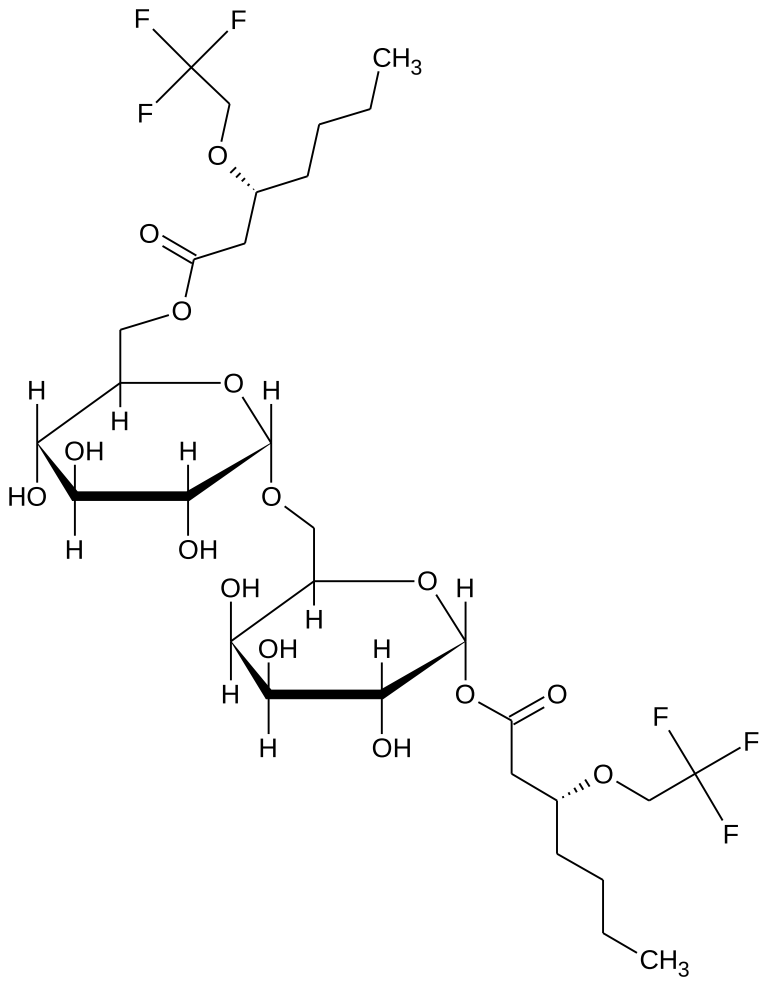


s) **F-mPHN-lac**: mixture of (3*R*)-3-(2,2,2-trifluoroethoxy)nonanoate lactose and (3*R*)-3-(2,2,2-trifluoroethoxy)heptanoate lactose mono and diesters

**Fig 1. List of compound names, shortcuts and structures generated in ChemSketch**

**Table 1. Precursor and product ions of sugar esters obtained in MRM LC-MS QQQ analysis:**

| Compound: | | MRM: Precursor ion -> product ion | Fragmentor: | Collision energy: |
|---|---|---|---|---|
| C9-gluc | C9 monoester | [M+K]-: 359.14 -> 341.3 | 93 | 6 |
| | | [M+K]-: 359.14 ->193.3 | | 18 |
| | | [M+K]-: 359.14 -> 41.1 | | 74 |
| | | [M+K]-: 359.14 -> 202.5 | | 26 |
| | C9 C9 diester | [M+Na]+: 483.69 -> 69 | 142 | 22 |
| C9-gal: | C9 monoester | [M+K]-: 359.14 -> 341.3 | 98 | 6 |
| | | [M+K]-: 359.14 -> 43.1 | | 54 |
| | | [M+K]-: 359.14 -> 202.4 | | 18 |
| | C9 C9 diester | [M+Na]+: 483.69 -> 69 | 142 | 22 |
| C9-lac: | C9 monoester | [M+$H_2$O+H]-: 517.2 -> 157.1 | 136 | 22 |
| | | [M+$H_2$O+H]-: 517.2 -> 282.1 | | 46 |
| | C9 C9 diester | [M+Na]+: 657.3 -> 157.1 | 131 | 30 |
| mPHN- glu: | C9 monoester | [M+Cl]-: 371,15 -> 35 | 98 | 14 |
| | C7 monoester | [M+K]+: 429.11 -> 73.1 | 132 | |
| | | [M+MeOH]+: 340.15 -> 295.7 | | 6 |
| | | [M+MeOH]+: 340.15 -> 180.4 | | 26 |
| | C9 C9 diester | [M+Cl]-: 527,26 -> 35 | 137 | 30 |
| | C7 C7 diester | - | - | - |
| | C9 C7 diester | [M+Cl]-: 499.23 -> 35 | 137 | 18 |
| mPHN- gal: | C9 monoester | [M+Cl]-: 371.15 -> 35 | 98 | 14 |
| | C7 monoester | [M+K]+: 429.11 -> 73.1 | | |
| | C9 C9 diester | [M+H]+: 493.3 -> 59.1 | 113 | 18 |
| | | [M+H]+: 493.3 -> 42.1 | | 126 |
| | C7 C7 diester | [M+H]+: 437.24 -> 23.1 | 181 | 30 |
| mPHN -lac: | C9 monoester | [M+H2O+H]-: 517.2 -> 157.1 | 116 | 26 |
| | | 517.2 -> 282.1 | 116 | 38 |
| | C9 C9 diester | [M+Na]+: 657.3 -> 157.1 | 111 | 26 |
| F-mPHN-gluc: | C9 monoester | [M+K]+: 457.14 -> 23 | 172 | 34 |
| | C7 monoester | [M+K]:+429.11 -> 73.1 | 245 | 46 |
| | | [M+K]:+429.11 -> 45 | | 94 |
| | | [M+K]:+429.11 -> 43.1 | | 178 |
| | | [M+K]:+429.11 -> 218.7 | | 22 |
| | C9 C9 diester wth two –$CF_3$ | [M+MeOH]+: 688.3 -> 73.1 | 147 | 70 |
| | | [M+MeOH]+: 688.3 -> 355.1 | | 26 |
| | C9 C7 diester with one –$CF_3$ | [M+Na]+: 651.26 -> 73 | 235 | 78 |
| | | [M+Na]+: 651.26 -> 45.1 | | 166 |
| | C9 C7 diester with one –$CF_3$ | [M-H]-: 627.26 -> 325 | 191 | 38 |
| F-mPHN-gal: | C9 C7 diester with two –$CF_3$ | [M+H]+: 629.28 -> 324.4 | 113 | 14 |
| | C7 C7 diester with one –$CF_3$ group | [M+H]+: 457.14 -> 23.1 | 167 | 38 |
| F-mPHN-lac: | C9 C9 diester with two –$CF_3$ groups | [M+MeOH]+: 851.36 -> 73.1 | 162 | 114 |
| | | [M+MeOH]+: 851.36 -> 436.4 | | 50 |
| | C9 C9 diester with one –$CF_3$ group | [M+Na]+: 759.34 -> 73.1 | 127 | 78 |
| | | [M+Na]+: 759.34 -> 45 | | 190 |
| | C7C7diester with one -$CF_3$ | [M+Na]+: 703.28 -> 355.1 | 118 | 30 |
| | C7C7 with two –$CF_3$ groups | [M+Na]+: 833.28 -> 73.1 | 181 | 122 |
| | | [M+Na]+: 833.28 -> 45 | | 198 |

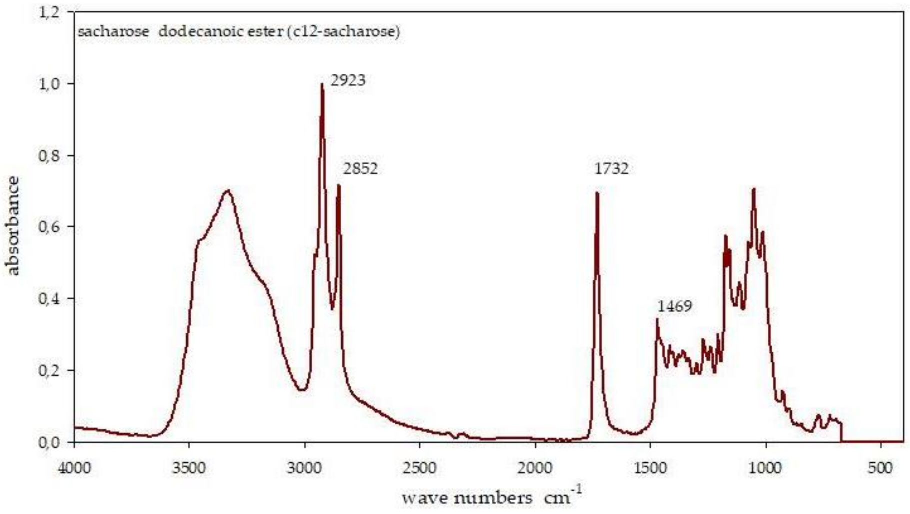


**Fig 2**. IR spectrum of C12- (sucrose monolaurate) as a referring compound

<u>Following peaks inform about:</u>

- Stretching vibrations: 3000- 3500 $cm^{-1}$ indicate presence -OH group
- Stretching vibrations: 2900 $cm^{-1}$ come from $-CH_2$ aliphatic residues
- Stretching vibrations around 1725 $cm^{-1}$ responsible for ester bonds

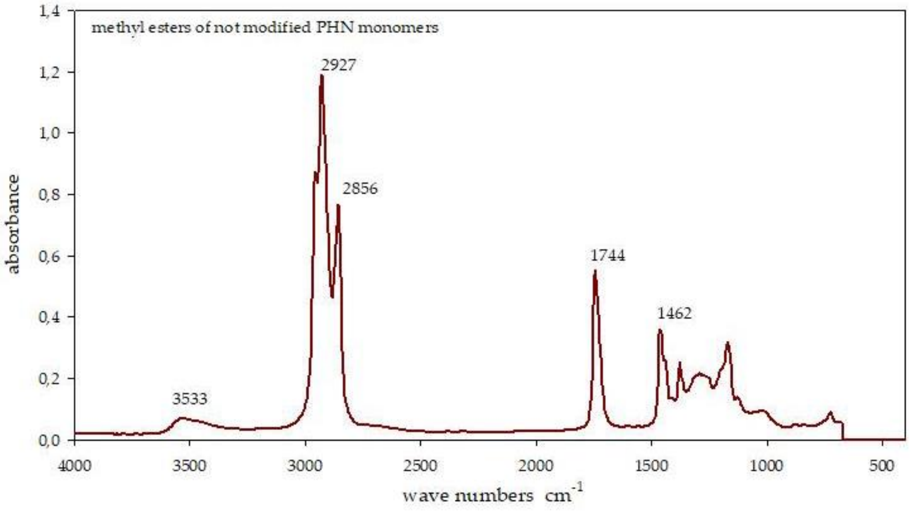


**Fig 3.** IR spectra of methyl esters of not modified PHN monomers (mixture of 3-(*R*)- hydroxynonanoic and 3-(*R*)- hydroxyheptanoic acids)

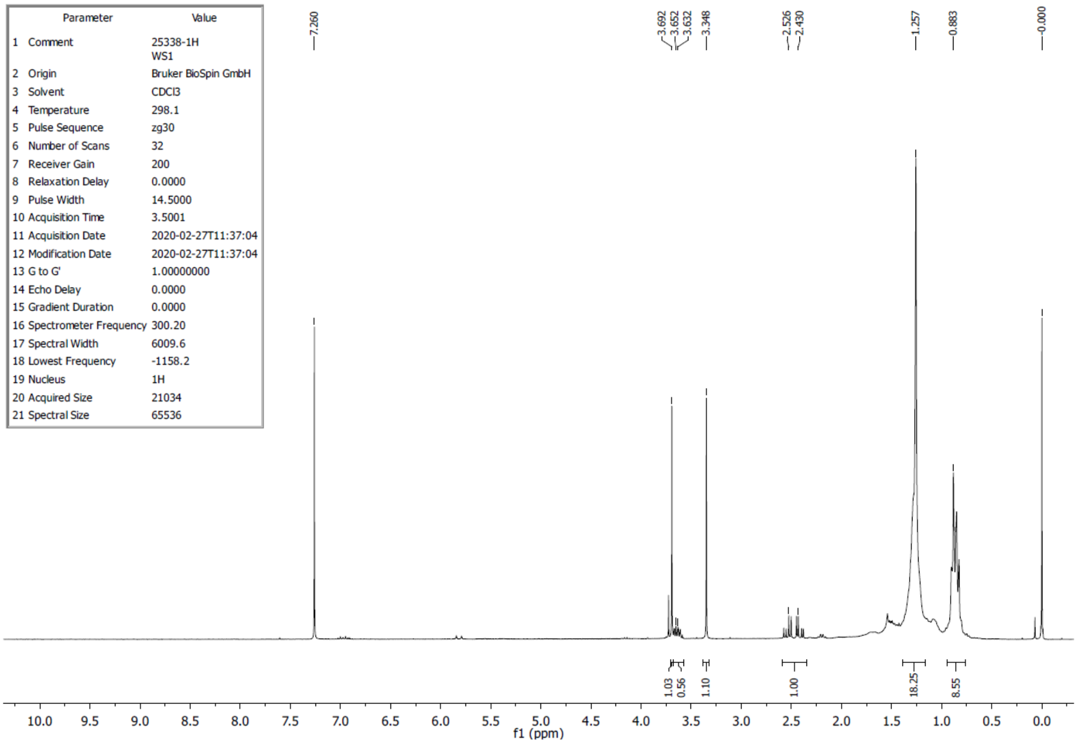


| | Parameter | Value |
|---|---|---|
| 1 | Comment | 25338-1H WS1 |
| 2 | Origin | Bruker BioSpin GmbH |
| 3 | Solvent | CDCl3 |
| 4 | Temperature | 298.1 |
| 5 | Pulse Sequence | zg30 |
| 6 | Number of Scans | 32 |
| 7 | Receiver Gain | 200 |
| 8 | Relaxation Delay | 0.0000 |
| 9 | Pulse Width | 14.5000 |
| 10 | Acquisition Time | 3.5001 |
| 11 | Acquisition Date | 2020-02-27T11:37:04 |
| 12 | Modification Date | 2020-02-27T11:37:04 |
| 13 | G to G' | 1.00000000 |
| 14 | Echo Delay | 0.0000 |
| 15 | Gradient Duration | 0.0000 |
| 16 | Spectrometer Frequency | 300.20 |
| 17 | Spectral Width | 6009.6 |
| 18 | Lowest Frequency | -1158.2 |
| 19 | Nucleus | 1H |
| 20 | Acquired Size | 21034 |
| 21 | Spectral Size | 65536 |

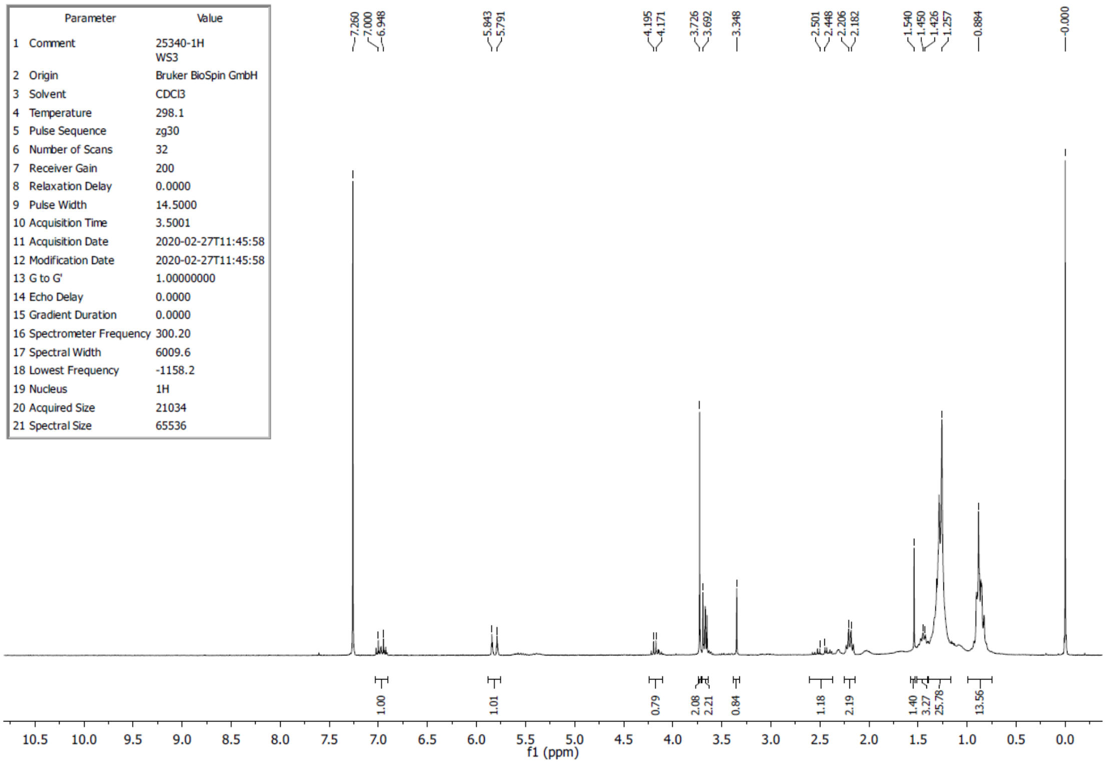


| | Parameter | Value |
|---|---|---|
| 1 | Comment | 25340-1H WS3 |
| 2 | Origin | Bruker BioSpin GmbH |
| 3 | Solvent | CDCl3 |
| 4 | Temperature | 298.1 |
| 5 | Pulse Sequence | zg30 |
| 6 | Number of Scans | 32 |
| 7 | Receiver Gain | 200 |
| 8 | Relaxation Delay | 0.0000 |
| 9 | Pulse Width | 14.5000 |
| 10 | Acquisition Time | 3.5001 |
| 11 | Acquisition Date | 2020-02-27T11:45:58 |
| 12 | Modification Date | 2020-02-27T11:45:58 |
| 13 | G to G' | 1.00000000 |
| 14 | Echo Delay | 0.0000 |
| 15 | Gradient Duration | 0.0000 |
| 16 | Spectrometer Frequency | 300.20 |
| 17 | Spectral Width | 6009.6 |
| 18 | Lowest Frequency | -1158.2 |
| 19 | Nucleus | 1H |
| 20 | Acquired Size | 21034 |
| 21 | Spectral Size | 65536 |

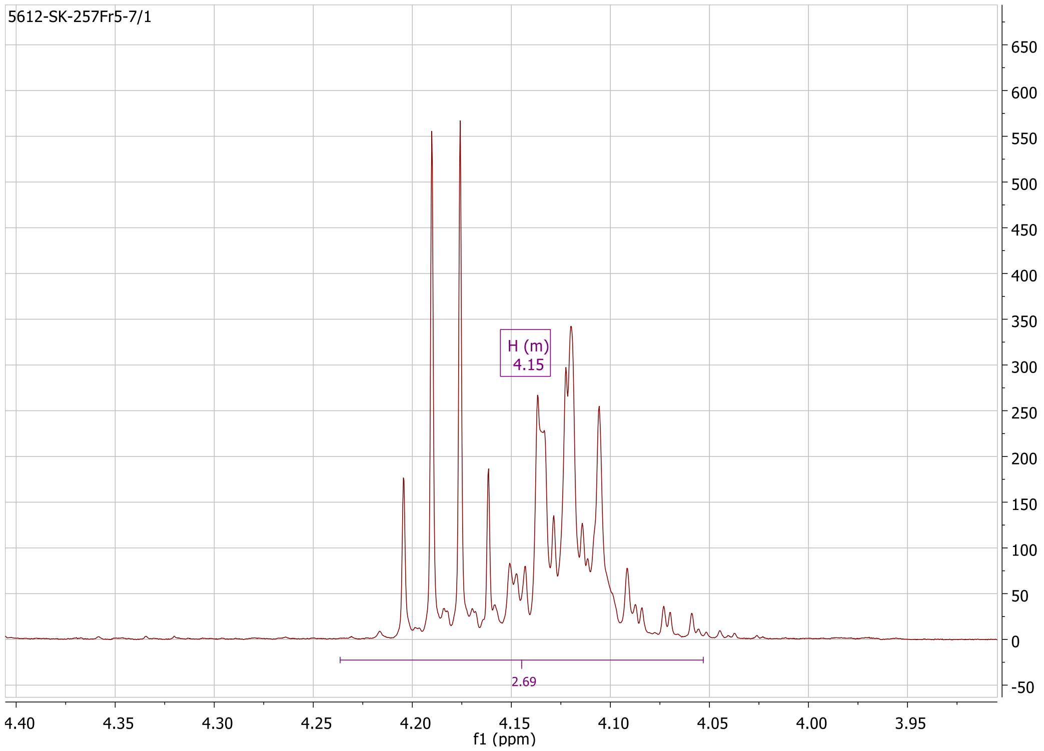


a) $^{1}$HNMR spectra

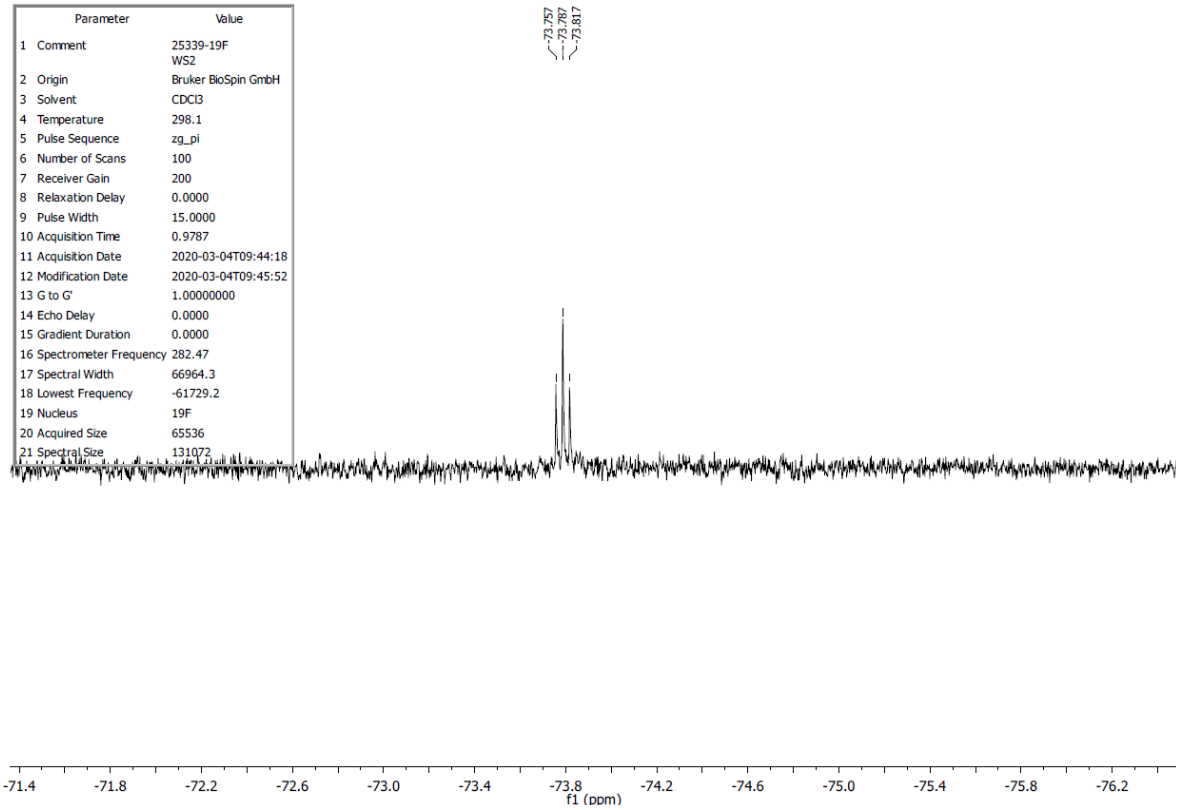


b) $^{19}$F NMR spectra of the modified PHN monomers confirmed a presence of fluoric moieties

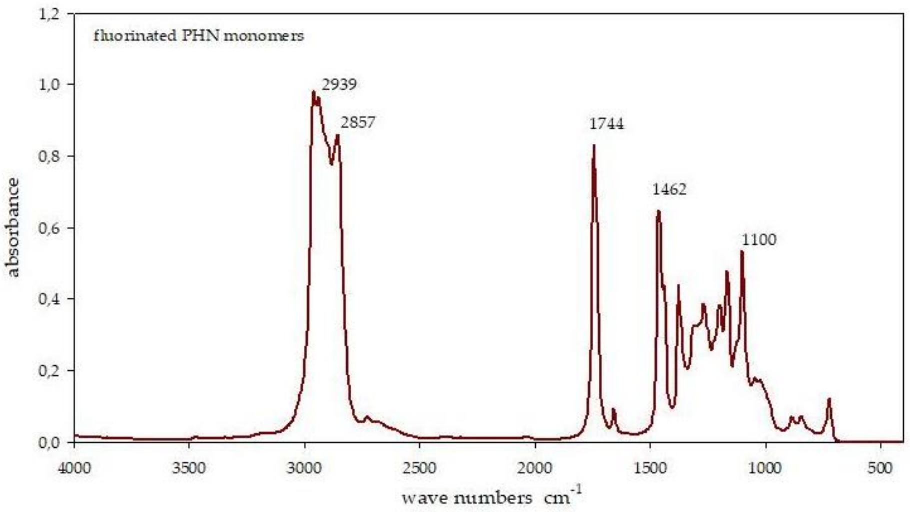


c) IR spectra of the purified PHN mixture after modification

**Fig 4.** Spectra analysis of methyl esters of methyl esters of 3-(*R*)- (2 -2′- 2′′- trlifluoroethyl)-nonanoic and heptanoic acids (fluorinated PHN monomers or mPHN-F)

**IR Spectra description:**

Stretching vibrations between ~ 1360-1090 $cm^{-1}$ suggests presence of -$CF_3$ groups

Stretching vibrations between ~ 1360-1000 $cm^{-1}$ and 1110-1000 $cm^{-1}$ suggests presence of –CF bonds

And 2859 $cm^{-1}$ for -C-O-C etheric bonds

1734 $cm^{-1}$ stretching vibrations inform about presence of ester bonds between methyl groups and carboxyl groups of PHN monomers

Spectrum profile refers to inverted IR spectra of 2,*2*,*2*-Trifluoroethyl Methyl Ether taken from*:* *"Low-Resolution Microwave, Infrared, and Raman Spectra, Conformational Stability, and Vibrational Assignment of 2,2,2-Trifluoroethyl Methyl Ether"*; J. Phys. Chem. 1987, 91, 1334-1344

Additionally, lack of large peak between 3000-3500 $cm^{-1}$ suggests –OH groups are not present in a structure of the analyzed compound which confirms that reaction of protecting –OH group occurred

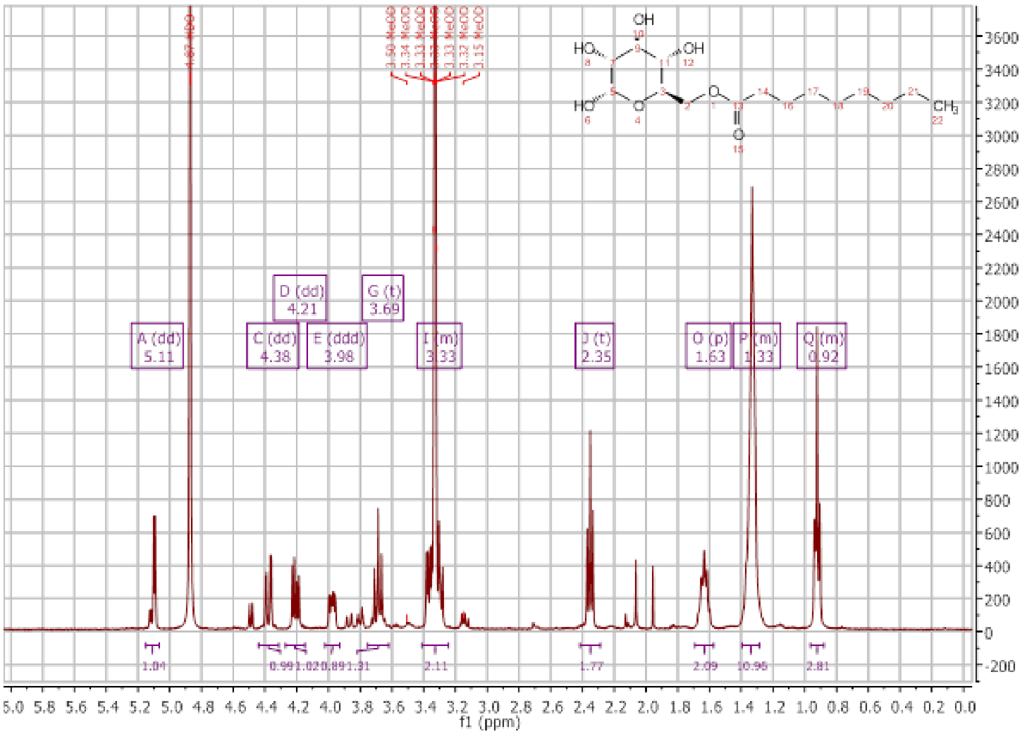


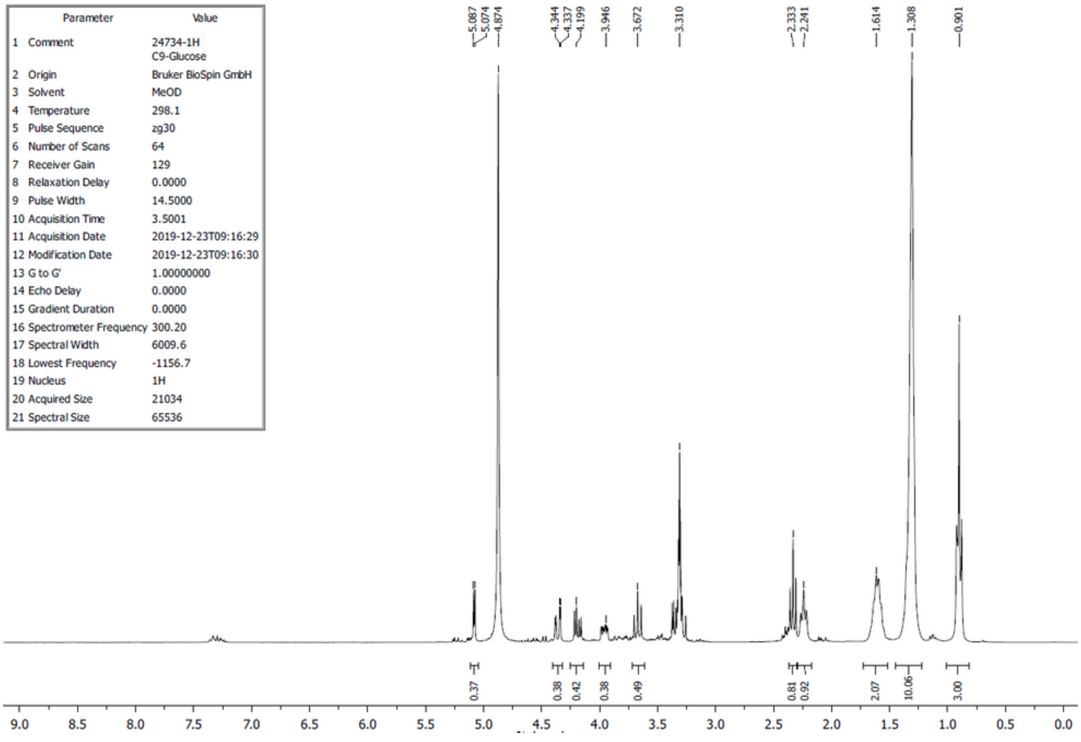


a) $^{1}$HNMR spectra

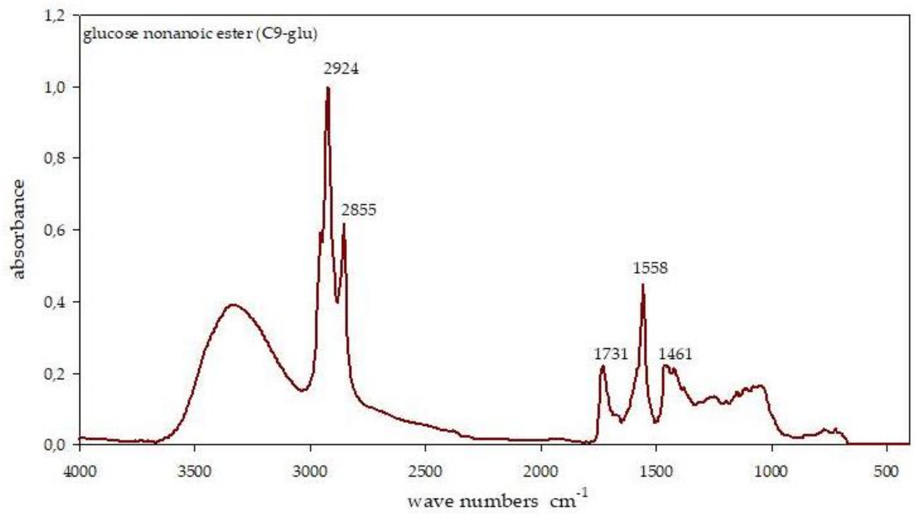


b) IR spectra

**Fig 5**. Spectral analysis of glucose nonanoate (C9-glu)

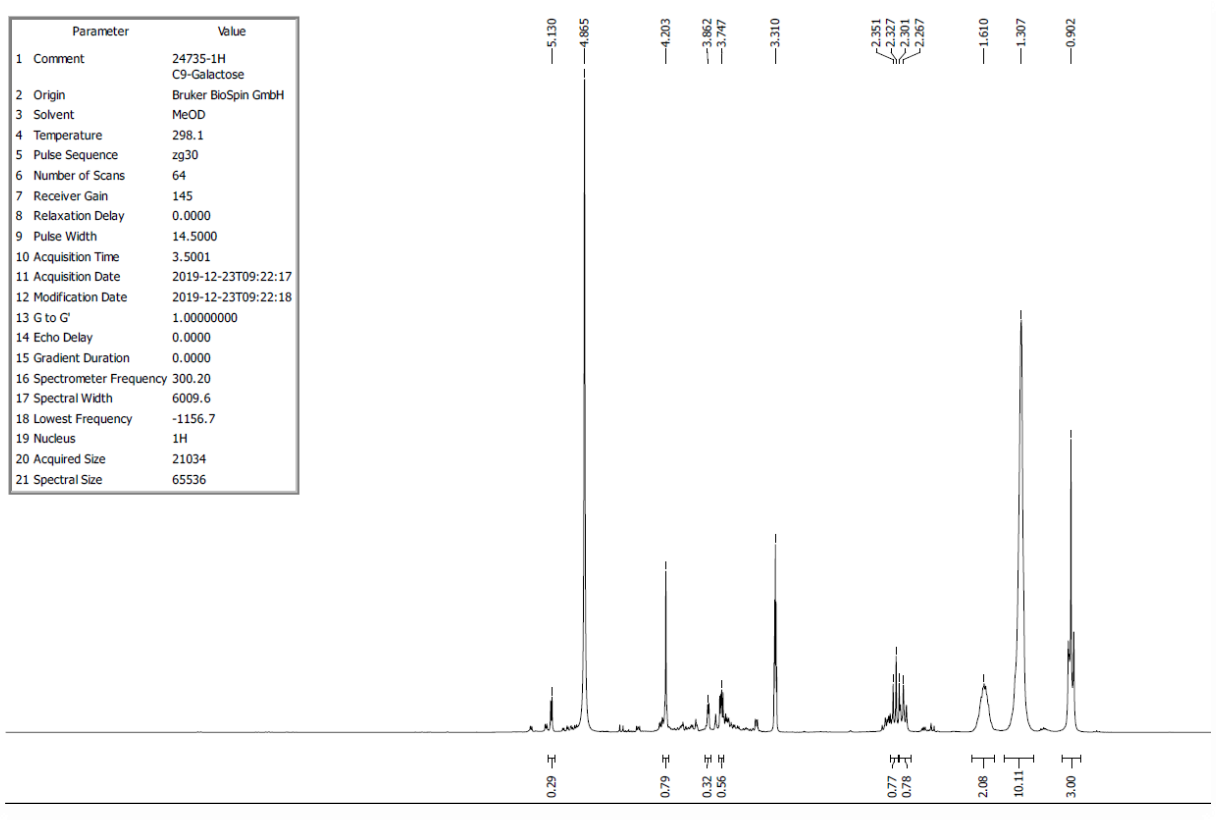

a) ¹HNMR spectra

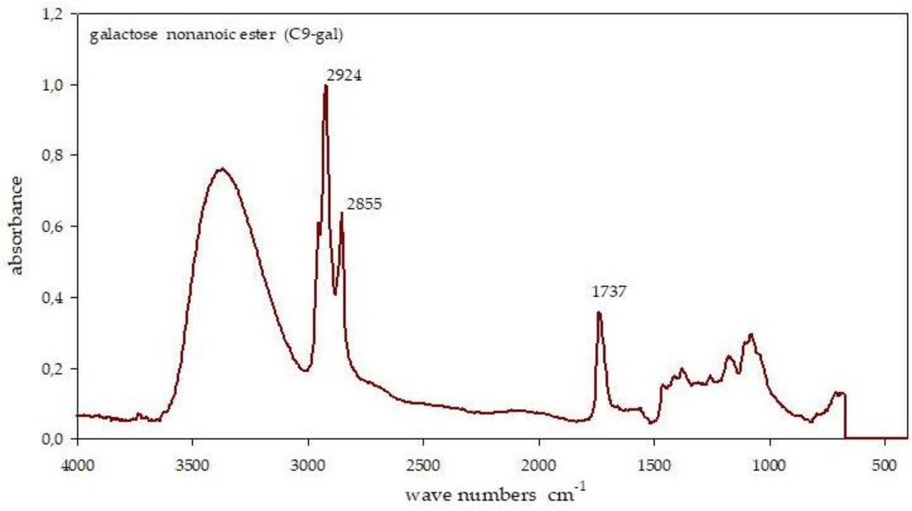


a) IR spectra

**Fig 6.** Spectral analysis galactose nonanoate (C9-gal)

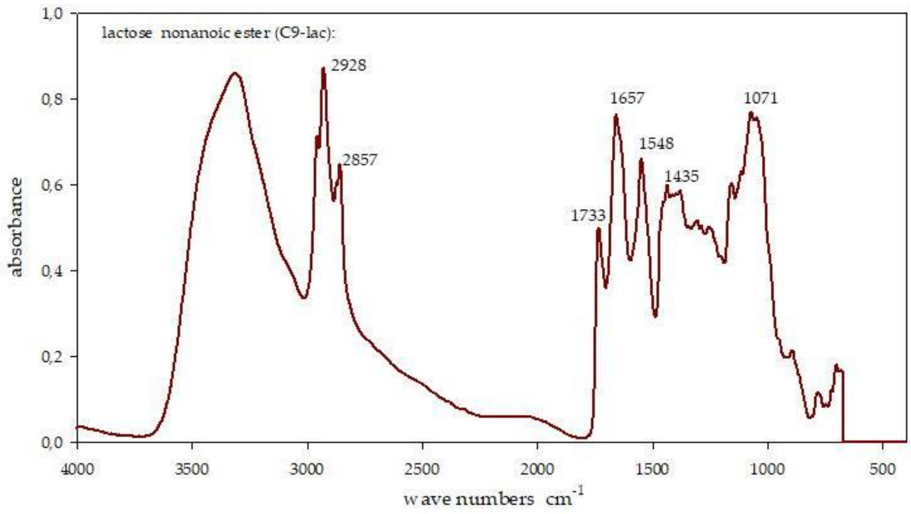


**Fig 7.** IR spectra of lactose nonanoate (C9-lac)

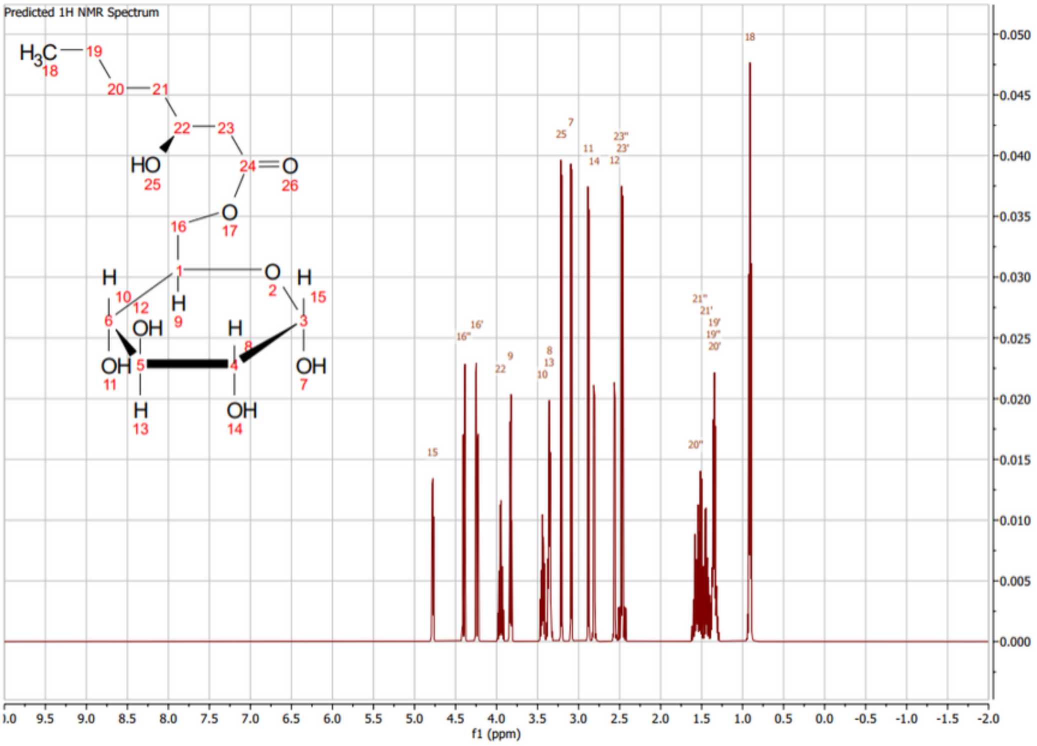


a) ¹HNMR spectra prediction

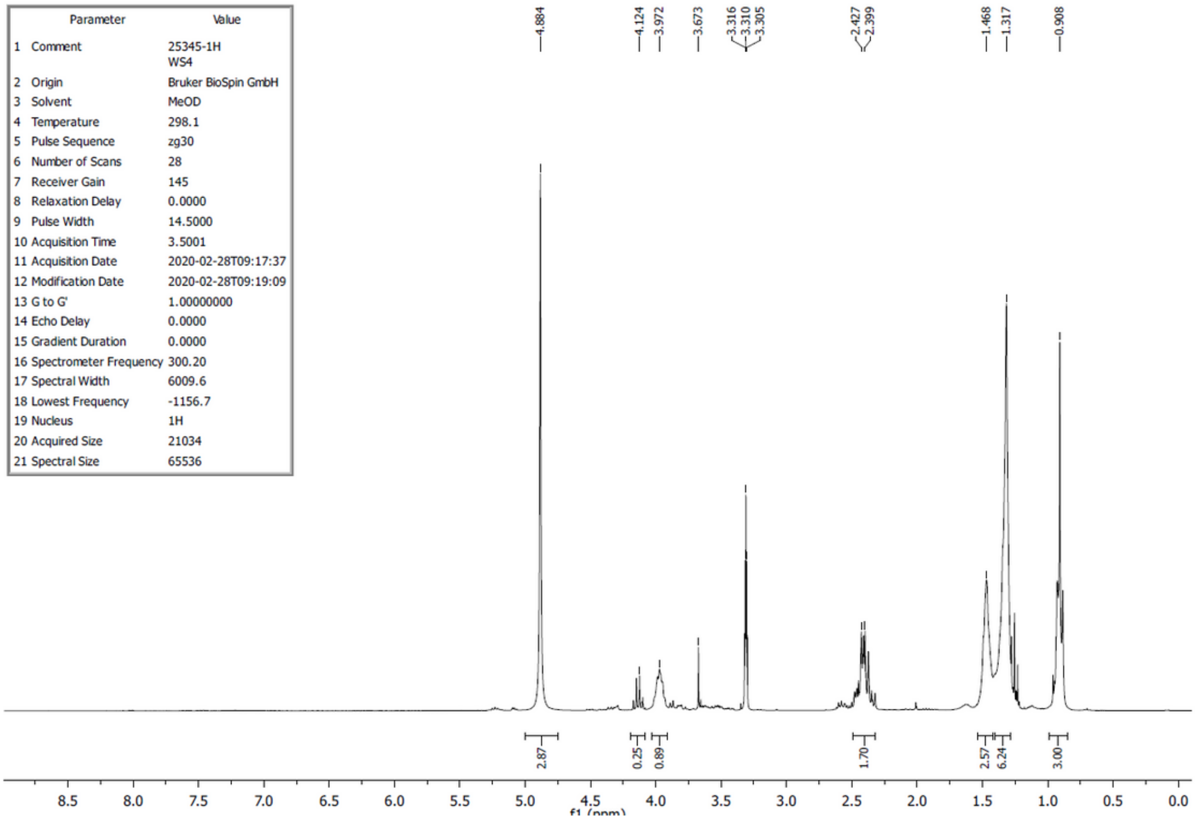


b) ¹HNMR spectra: ¹H NMR (300 MHz, Methanol-d4) δ 4.93 (s, 3H), 4.10 – 3.89 (m, 1H), 3.33 (td, *J* = 3.7, 2.1 Hz, 1H), 2.55 – 2.29 (m, 2H), 1.62 – 1.21 (m, 12H), 1.02 – 0.85 (m, 3H)

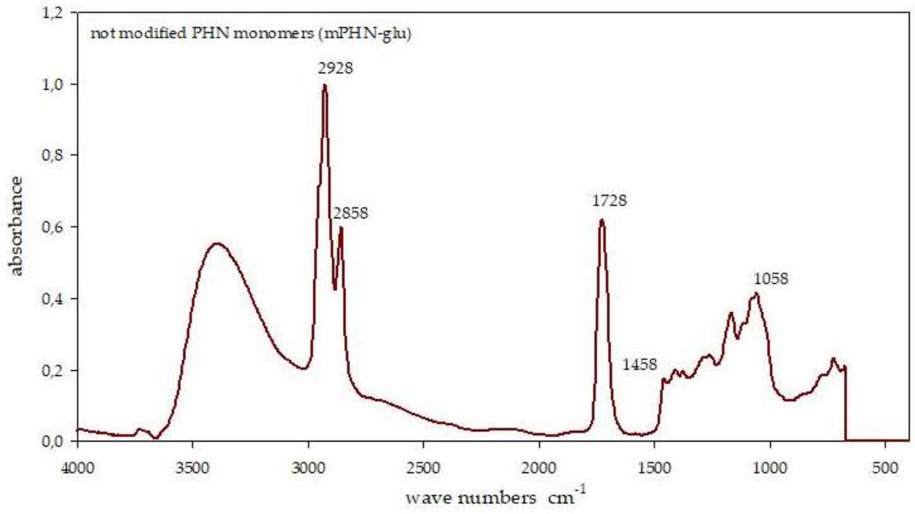


c) IR spectra

**Fig 8.** Spectral analysis of glucose mono and diesters esters originated from mixture of not modified PHN monomers (mPHN-glu)

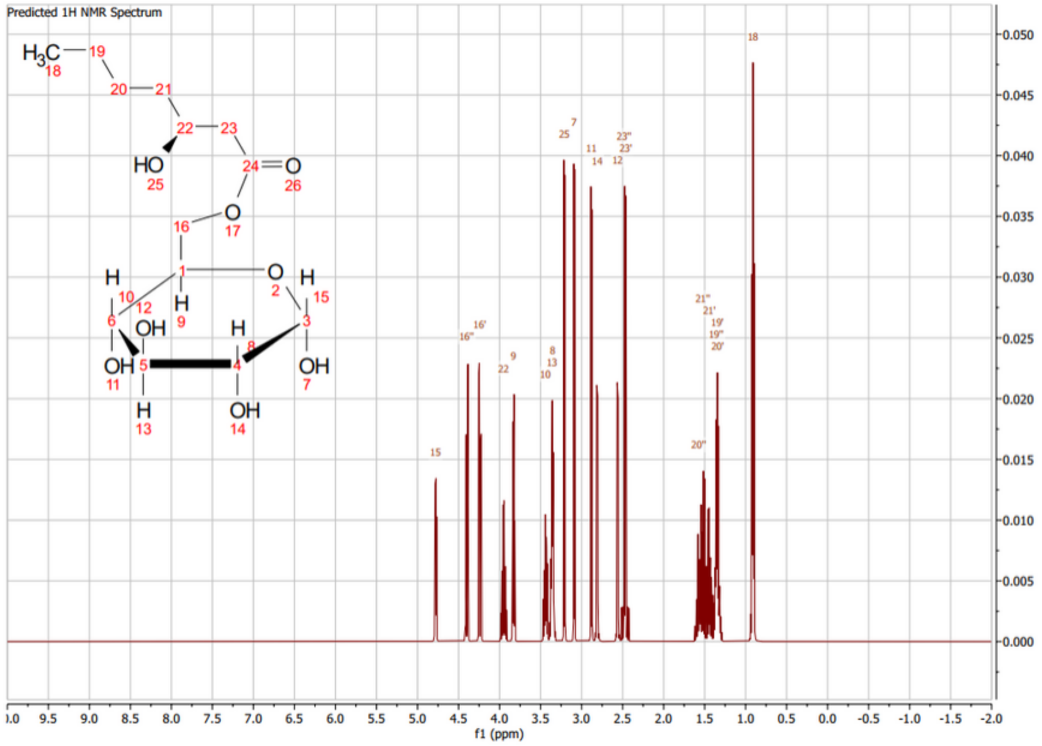


a) $^{1}$HNMR spectra prediction

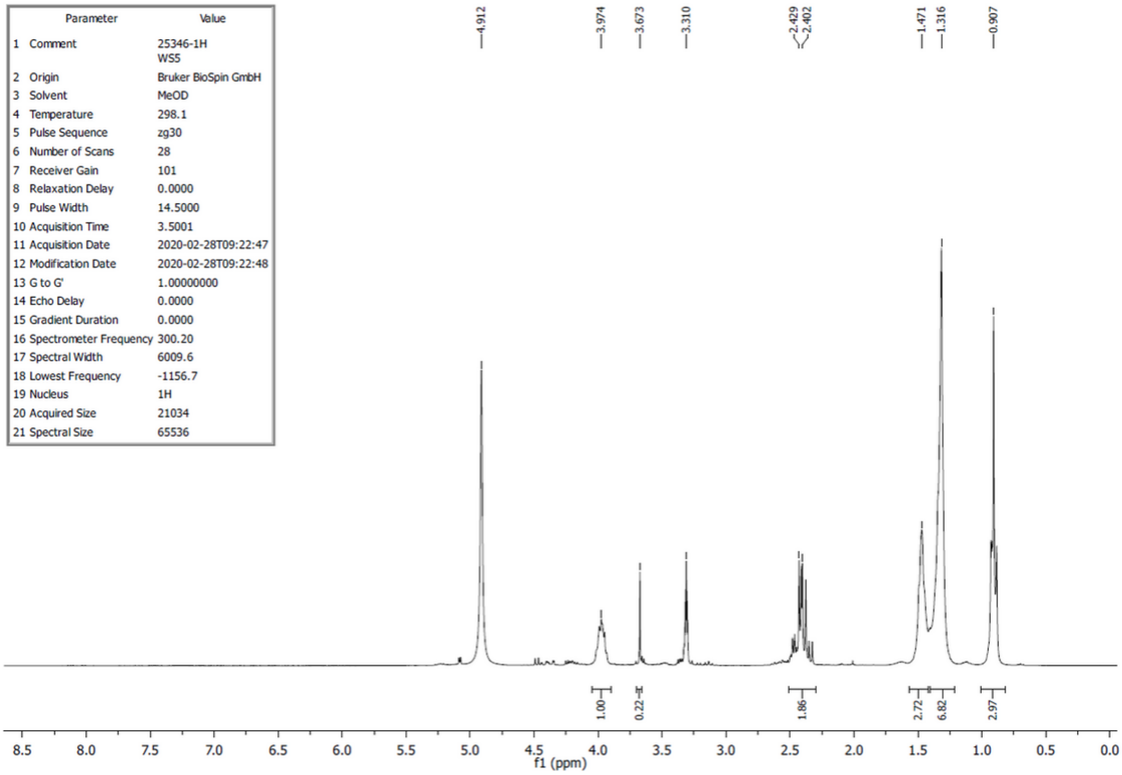


b) $^{1}$HNMR spectra: $^{1}$H NMR (300 MHz, Methanol-d4) δ 4.94 (s, 3H), 3.99 (dtd, *J* = 9.5, 7.0, 3.4 Hz, 1H), 3.33 (p, *J* = 1.7 Hz, 1H), 2.54 – 2.28 (m, 2H), 1.63 – 1.22 (m, 12H), 1.03 – 0.84 (m, 3H).

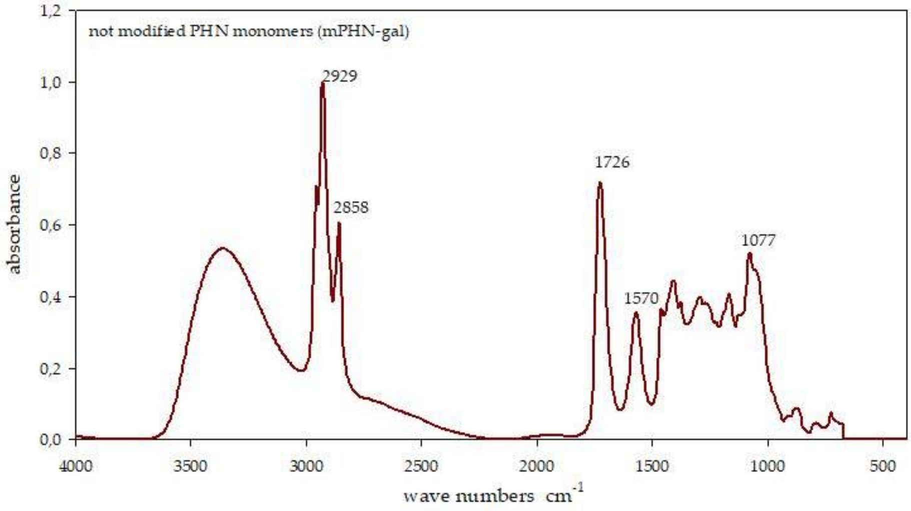


c) IR spectra

**Fig 9.** Spectral analysis of galactose mono and diesters esters originated from mixture of not modified PHN monomers (mPHN-gal)

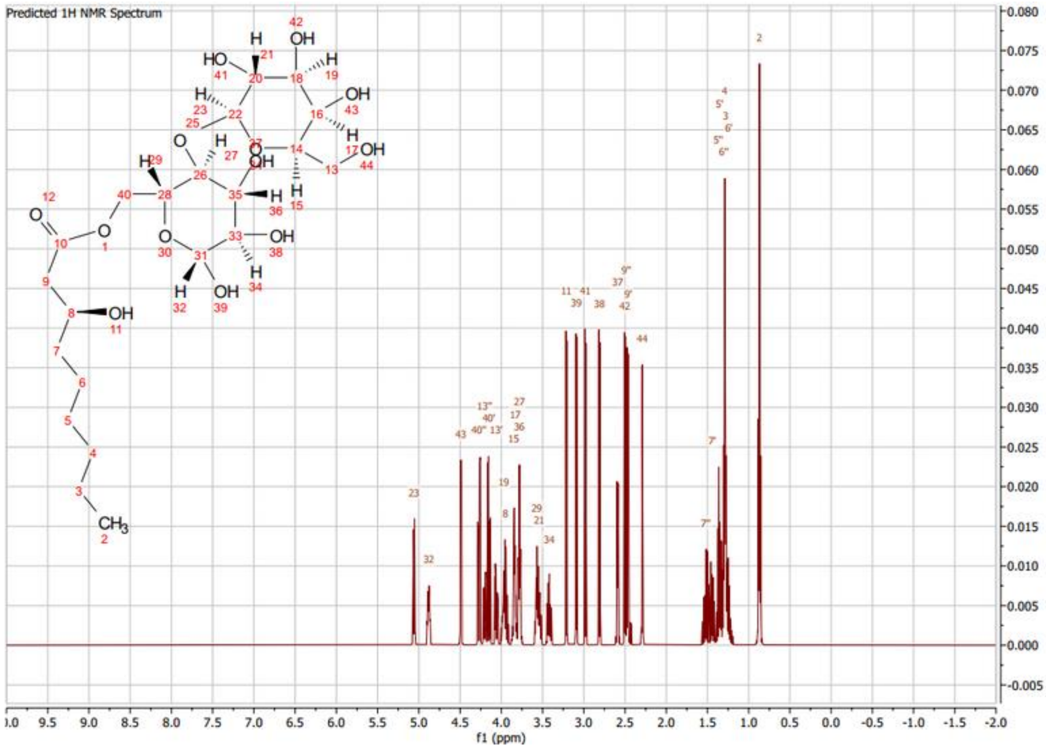

a) $^{1}$HNMR spectra prediction

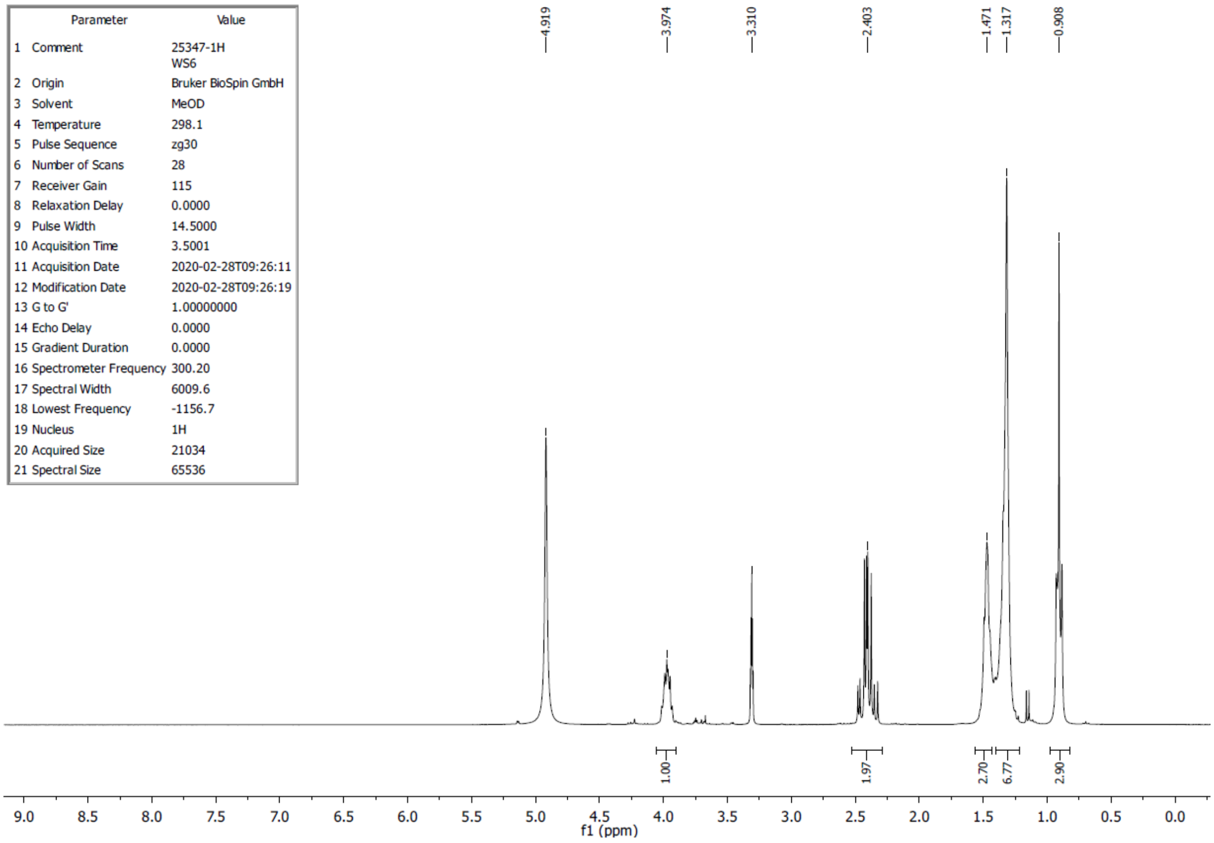


b) $^{1}$HNMR spectra: (300 MHz, Methanol-d4) δ 4.90 (s, 3H), 4.16 (q, *J* = 7.1 Hz, 1H), 4.07 – 3.91 (m, 1H), 3.33 (p, *J* = 1.6 Hz, 1H), 2.55 – 2.31 (m, 2H), 1.58 – 1.23 (m, 11H), 1.03 – 0.82 (m, 3H)

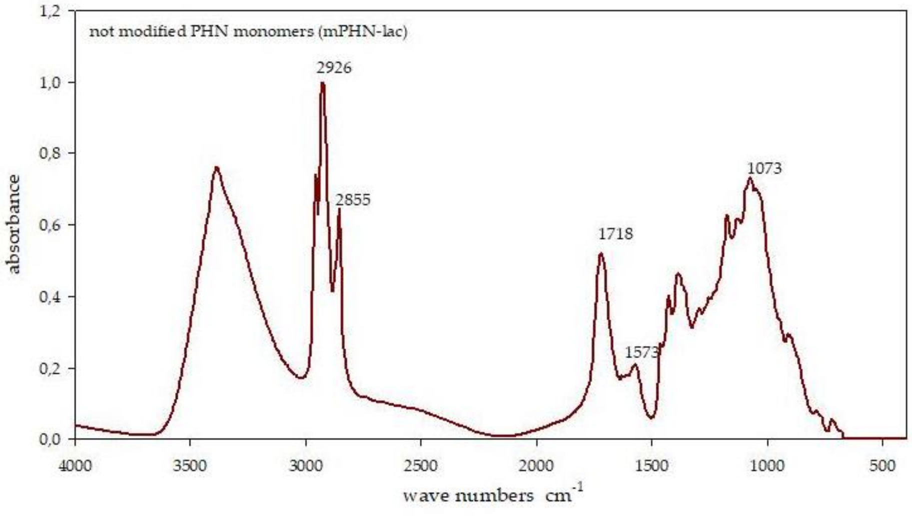

c) IR spectra

**Fig 10.** Spectral analysis of lactose mono and diesters esters originated from mixture of not modified PHN monomers (mPHN-lac)

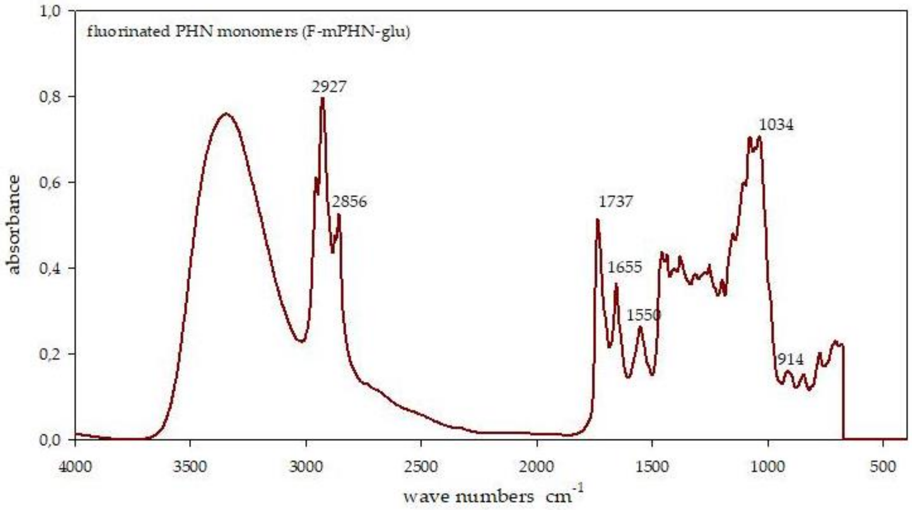


**Fig 11.** IR spectra of glucose mono and diesters esters originated from mixture of fluorinated PHN monomers (F-mPHN-glu)

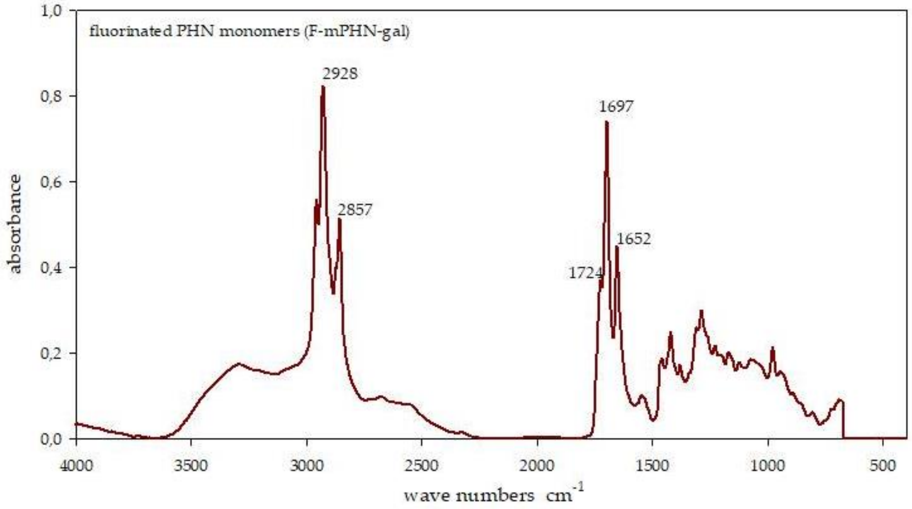

**Fig 12.** IR spectra of galactose mono and diesters esters originated from mixture of fluorinated PHN monomers (F-mPHN-gal)

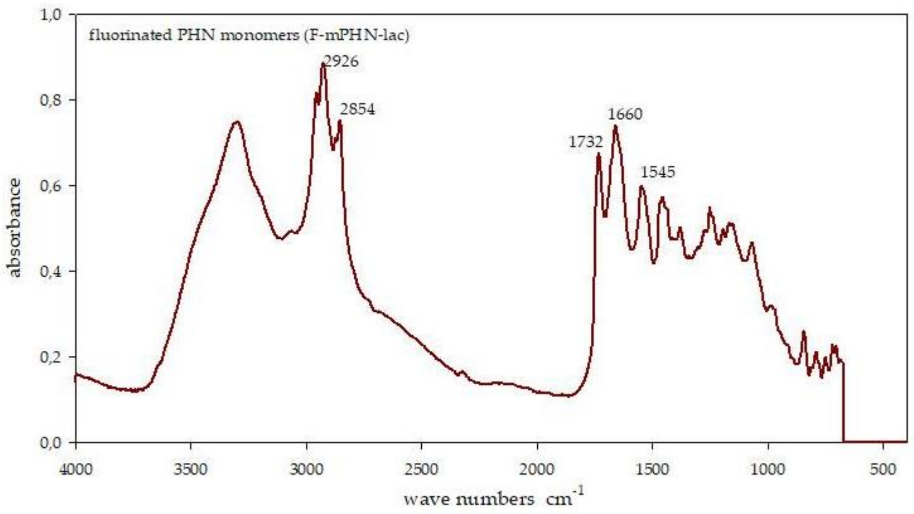


**Fig 13.** IR spectra of lactose mono and diesters esters originated from mixture of fluorinated PHN monomers (F-mPHN-lac)

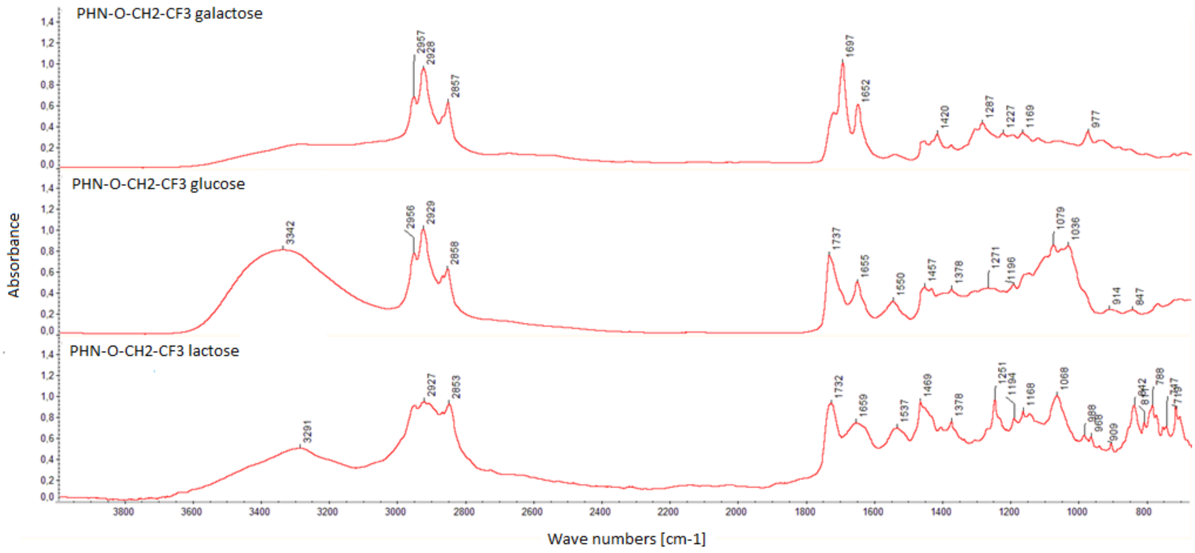


**Fig 14.** IR spectra of fluorinated esters set together

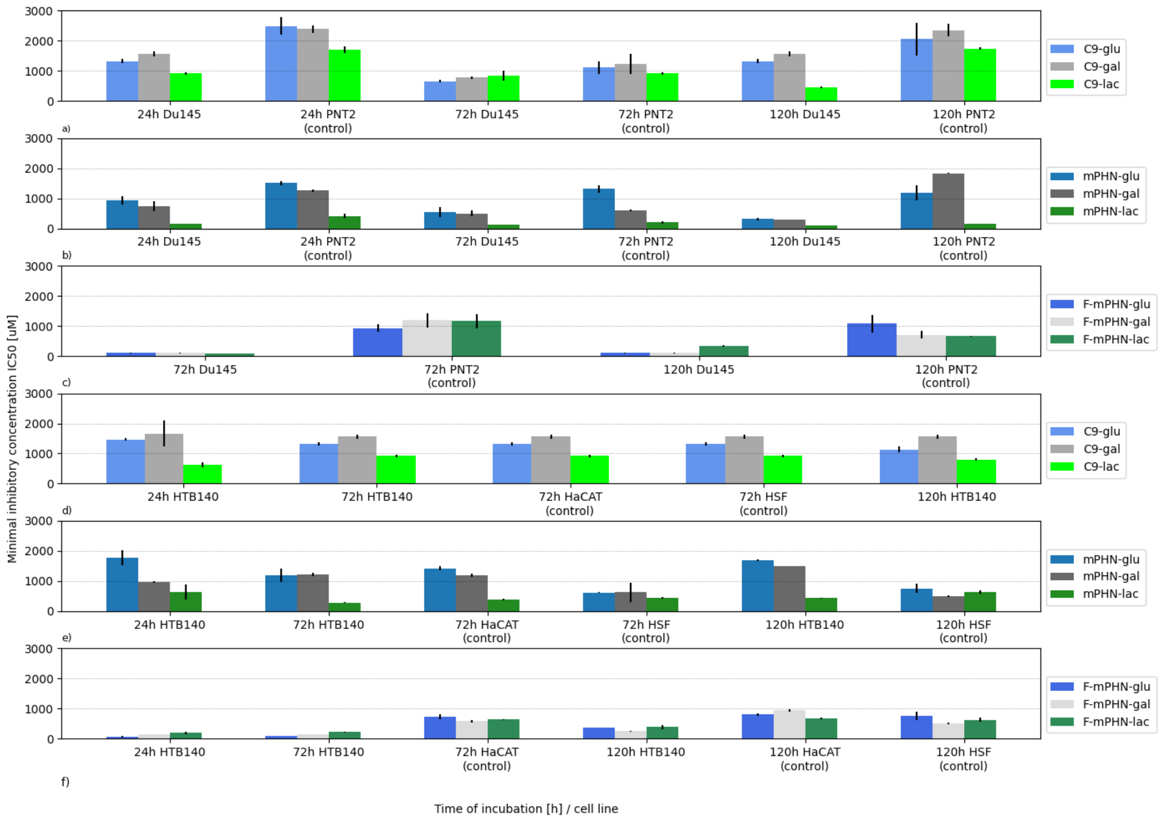


**Fig 15**. Cytotoxicity of SFAE determined by MTT assay, where $IC_{50}$ is a concentration [μM] of a particular compound that causes reduction of a tested cell culture to 50% :

a) nonanoic acid originated SFAE tested on DU145 / PNT2 (control)

b) mPHN originated SFAE tested on DU145 / PNT2 (control)

c) F-mPHN originated SFAE tested on DU145 / PNT2 (control)

d) nonanoic acid originated SFAE tested on HTB40 /HaCat/ HSF (control)

e) mPHN originated SFAE tested on HTB40 /HaCat/ HSF (control)

f) F-mPHN originated SFAE tested on HTB40 /HaCat/ HSF (control)

**Table 2.** Cytotoxicity of SFAE determined by MTT assay: – not determined; d – standard deviation. Red fillings mark the most lowest concentrations, green- the highest.

| | Time [h] | C9-glu | C9-gal | C9-lac | PHN-glu | PHN-gal | PHN-lac | F-mPHN-glu | F-mPHN-gal | F-mPHN-lac |
|---|---|---|---|---|---|---|---|---|---|---|
| DU145 (prostate cancer) | 24 | 1317.1 | 1560.5 | 919.5 | 932.1 | 750.9 | 161.2 | - | - | - |
| | d | 66 | 78 | 46 | 146 | 169 | 3 | - | - | - |
| | 72 | 658.6 | 780.3 | 836.9 | 541.1 | 500.0 | 131.4 | 100.0 | 100.0 | 83.8 |
| | d | 33 | 39 | 169 | 159 | 100 | 5 | 5 | 5 | 4 |
| | 120 | 1317.1 | 1560.8 | 459.8 | 315.7 | 304.6 | 91.3 | 100.0 | 100.0 | 335.0 |
| | d | 66 | 78 | 23 | 41 | 2 | 3 | 5 | 5 | 17 |
| PNT2 (control) | 24 | 2491.5 | 2390.9 | 1707.2 | 1507.6 | 1256.8 | 421.0 | - | - | - |
| | d | 285 | 120 | 110 | 80 | 32 | 79 | - | - | - |
| | 72 | 1110.5 | 1233.3 | 919.5 | 1312.2 | 612.5 | 199.5 | 926.8 | 1183.5 | 1156.4 |
| | d | 207 | 327 | 46 | 115 | 31 | 42 | 127 | 238 | 234 |
| | 120 | 2053.1 | 2355.3 | 1743.7 | 1180.2 | 1838.4 | 165.2 | 1075.5 | 704.9 | 662.5 |
| | d | 538 | 220 | 33 | 247 | 20 | 5 | 296 | 117 | 8 |
| HTB 140 (melanoma) | 24 | 1468.8 | 1659.0 | 621.6 | 1767.6 | 967.3 | 640.0 | 63.3 | 155.9 | 189.0 |
| | d | 46 | 434 | 77 | 253 | 26 | 250 | 24 | 3 | 49 |
| | 72 | 1317.1 | 1560.5 | 919.5 | 1195.7 | 1224.9 | 283.4 | 89.3 | 155.9 | 216.9 |
| | d | 66 | 78 | 46 | 232 | 61 | 8 | 2 | 3 | 21 |
| | 120 | 1133.9 | 1560.5 | 805.3 | 1689.4 | 1496.4 | 435.0 | 372.4 | 247.9 | 394.2 |
| | d | 92 | 78 | 51 | 35 | 2 | 10 | 3 | 11 | 70 |
| HaCAT (human keratinocytes-control) | 72 | 1317.1 | 1560.5 | 919.5 | 1427.3 | 1200.0 | 383.9 | 733.7 | 580.0 | 644.1 |
| | d | 66 | 78 | 46 | 71 | 60 | 25 | 81 | 37 | 17 |
| | 120 | - | - | - | - | - | - | 800.8 | 945.9 | 670.0 |
| | d | - | - | - | - | - | - | 40 | 47 | 33 |
| HSF (human skin fibroblasts-control | 72 | 1317.1 | 1560.5 | 919.5 | 613.4 | 624.2 | 445.8 | - | - | - |
| | d | 66 | 78 | 46 | 12 | 322 | 24 | - | - | - |
| | 120 | - | - | - | 761.2 | 499.6 | 631.1 | 761.2 | 499.6 | 631.1 |
| | d | - | - | - | 143 | 25 | 64 | 143 | 25 | 64 |
